# A Novel Cognitive Frailty Index for Geriatric Mice

**DOI:** 10.1111/acel.70056

**Published:** 2025-05-21

**Authors:** Serena Marcozzi, Giorgia Bigossi, Maria Elisa Giuliani, Giovanni Lai, Beatrice Bartozzi, Marta Balietti, Tiziana Casoli, Fiorenza Orlando, Andrea Amoroso, Robertina Giacconi, Maurizio Cardelli, Francesco Piacenza, Fabrizia Lattanzio, Fabiola Olivieri, Peter L. J. de Keizer, Fabrizio d’Adda di Fagagna, Marco Malavolta

**Affiliations:** ^1^ Advanced Technology Center for Aging Research and Geriatric Mouse Clinic IRCCS INRCA Ancona Italy; ^2^ Advanced Technology Center for Aging Research IRCCS INRCA Ancona Italy; ^3^ Center for Neurobiology of Aging IRCCS INRCA Ancona Italy; ^4^ Experimental Animal Models for Aging Unit Scientific Technological Area, IRCCS INRCA Ancona Italy; ^5^ Charles River Laboratories Calco Italy; ^6^ Scientific Direction IRCCS INRCA Ancona Italy; ^7^ Department of Clinical and Molecular Sciences DISCLIMO, Università Politecnica Delle Marche Ancona Italy; ^8^ Center for Molecular Medicine, Division of Laboratories, Pharmacy and Biomedical Genetics University Medical Center Utrecht Utrecht the Netherlands; ^9^ Cleara Biotech B.V. Utrecht the Netherlands; ^10^ IFOM ETS—The AIRC Institute of Molecular Oncology Milan Italy; ^11^ Institute of Molecular Genetics IGM‐CNR “Luigi Luca Cavalli‐Sforza” Pavia Italy

**Keywords:** cognitive aging, cognitive frailty index, Geroscience, longitudinal assessment, spatial performances

## Abstract

Loss of cognitive function is a significant challenge in aging, and developing models to understand and target cognitive decline is crucial for the development of Geroscience‐based interventions. Aged mice offer a valuable model as they share features of cognitive decline with humans. Despite numerous studies, knowledge of longitudinal age‐related cognitive changes and cognitive frailty in naturally aging mice is limited, particularly in cohorts exceeding 30 months of age, where cognitive decline is more pronounced. Moreover, the impaired physical function of aged mice is known to affect latency‐based strategies to measure cognitive performances. Here, we show a comprehensive longitudinal assessment using the Barnes Maze test in a large cohort of 424 aged (≥ 21 months) C57BL/6J mice. We introduced a new metric, the Cognitive Frailty Index (CoFI), which summarizes different age‐associated Barnes Maze parameters into a unique function. CoFI strongly associates with advancing age and mortality, offering a reliable ability to discriminate long‐ and short‐lived mice. We also established a CoFI cut‐off and a physically adjusted CoFI, both of which can distinguish between physical and cognitive frailty. This is further supported by the enhanced predictive power when physical and cognitive frailty are combined to assess short‐term mortality. Moreover, the computation method for CoFI is adaptable to various cognitive assessment tests, leveraging procedures akin to those used for calculating other frailty indices. In conclusion, through robust longitudinal tracking, CoFI has the potential to become an important ally in assessing the effectiveness of Geroscience‐based interventions to counteract age‐related cognitive impairment.

## Graphical abstract

Our study presents a groundbreaking longitudinal assessment of age‐related cognitive decline in a large cohort of aged mice. Through the development of the Cognitive Frailty Index (CoFI), we introduce a robust metric that correlates strongly with advancing age and mortality. Importantly, CoFI and physical frailty are distinct entities, with CoFI offering a new tool to assess cognitive impairment independently from physical decline. This adaptable tool will be valuable for testing the efficacy of innovative interventions in the field of Geroscience.

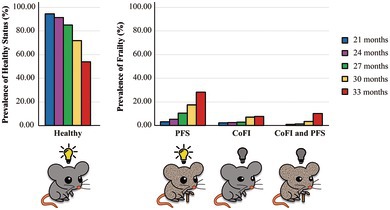

## 1 Introduction

Cognitive function decline with aging, impacting various domains such as conceptual reasoning, memory, and processing speed (Deary et al. 2009; Hedden and Gabrieli 2004; Hughes et al. 2018; Plassman et al. 2010; Wilson et al. 2002). This decline significantly reduces quality of life and is associated with an increased risk of disability and mortality (Buchman et al. 2013; Grande et al. 2020; Salthouse 2012; Wu et al. 2022). Therefore, preserving cognitive health represents a fundamental objective for fostering independent living for older adults and contributes to reducing the overall costs of long‐term care (Blazer et al. 2015; Coughlin and Liu 1989). However, conducting longitudinal studies on human aging is challenging and may take decades to be completed. These limitations highlight the need for alternative research options to study the effects of intervention aimed at reducing, potentially counteracting, the age‐related cognitive decline as well as to understand its molecular mechanisms. In this context, rodents offer several advantages as a preclinical model because of their ready availability and shorter lifespan, allowing a more detailed assessment of age‐related changes. Mice, as humans, experience cognitive decline with physiological aging (Brito et al. 2023; Radulescu et al. 2021). Numerous studies comparing young adult (2–4 months of age) and middle‐aged (8–15 months of age) animals revealed age‐related impairments in several cognitive domains, including learning, working memory, long‐ and short‐term memory, and cognitive flexibility (Chen et al. 2023; Ederer et al. 2022; Mishra and Thakur 2022; Shoji et al. 2016; Wang et al. 2020; Yanai and Endo 2021). Investigations involving old mice (18–24 months of age) and geriatric mice (28 months of age) demonstrate an even greater decline in cognitive function within these age cohorts (Daneshjoo et al. 2022; Ederer et al. 2022; Kushwaha and Thakur 2020; Pettan‐Brewer et al. 2013; Yanai and Endo 2021; Yang et al. 2019). Aged mice are also useful for assessing the impact on cognitive performance of any treatment aimed at mitigating or reversing aging, including those not designed to specifically target the central nervous system (Jayarathne et al. 2022). These considerations indicate mice as an appropriate model for studying human cognitive aging and for testing therapeutic approaches to slowing down or reversing cognitive decline and physiological functions. Nonetheless, despite numerous studies comparing young and aged mice, information on longitudinal age‐related cognitive changes remains limited, particularly in cohorts exceeding 30 months of age, where cognitive decline is more pronounced.

Developing an accurate and comprehensive method to assess cognitive deterioration associated with aging is therefore crucial. The potentially shared etiopathogenesis between cognitive and physical frailty is a crucial aspect of this topic that remains unresolved. Cognitive frailty in humans is characterized by the concurrent presence of physical frailty and cognitive impairment, in the absence of dementia, as defined by the International Academy of Nutrition and Aging and the International Association of Gerontology and Geriatrics (Dartigues and Amieva 2014; Sugimoto et al. 2022). However, it remains uncertain whether physical frailty and cognitive impairment should be considered: (1) a single phenotype, as proposed by the cognitive frailty construct; (2) distinct phenotypes arising from a common underlying mechanism; or (3) two independent conditions that often co‐occur in older adults. This issue continues to be an open question with no definitive answer based on current evidence.

Here, we introduce a novel scoring system, which we call Cognitive Frailty Index (CoFI), specifically designed to evaluate the longitudinal age‐related cognitive decline and to disentangle physical from cognitive frailty. This tool provides a potential standardized measurement of cognitive deterioration and holds the potential for assessing the efficacy of therapeutic interventions in geriatric mice.

## 2 Materials and Methods

### 2.1 Animals and Experimental Design

Four hundred twenty‐four C57BL/6J mice (48% males), mean age at the time of inclusion of 23.65 ± 1.31 months (males: 23.68 ± 1.32 months; females: 23.64 ± 1.30 months), housed in the Geriatric Mouse Clinic of IRCCS INRCA under Specific Pathogen Free conditions from 2020 to 2023, were enrolled in the study. All mice were control groups undergoing the same experimental procedures in various studies approved by the Italian Ministry of Health (authorization no. 1074/2020‐PR for 161 mice, authorization no. 137/2021‐PR for 23 mice, authorization no. 87/2022‐PR for 106 mice, and authorization no. 70/2023‐PR for 134 mice). Mice were housed at a maximum density of 6 per cage (standard cages with a floor area of 370 cm^2^) under a controlled environment, maintained on a 12:12 light–dark cycle (lights on at 6:00 a.m.), and had ad libitum access to food and water. To minimize the risk of aggression among male mice, animals housed together were littermates that had been co‐housed continuously since birth. This approach is widely recognized to reduce stress and fighting in male groups. Importantly, housing six male mice per cage was applied only in a limited number of cages and only when necessary to avoid mixing animals from different litters, which could increase the likelihood of aggression. Animal welfare was monitored daily by trained personnel, and no signs of fighting or injuries were observed during the study.

All mice underwent the same phenotypical assessments every three months, which included clinical frailty evaluations, physical performance tests, the Barnes Maze test, and the Novel Object Recognition (NOR) test. Nearly all mice (386; 176 males and 210 females) were longitudinally monitored until natural death, with only a small subset (censored in survival analysis) euthanized for organ explantation and subsequent biobanking purposes (56; 29 males and 27 females). This subset underwent at least two phenotypical assessments. The first measurement in all mice was performed between 21 and 27 months (Table S1).

If signs of distress or health deterioration were observed during the study, a detailed assessment was conducted based on predefined humane endpoint criteria. Further details on the assessment criteria and procedures can be found in the Appendix S1.

### 2.2 Novel Object Recognition Test

Recognition memory was assessed using the NOR test, adapted from Traschutz et al. (2018) and conducted during the daytime. The test consisted of three phases: habituation phase (1 day), training phase (1 day), and test phase (1 day).

During the habituation phase, each mouse was placed in an empty arena (35 × 35 cm) for 10 min. The following day, two identical objects were introduced for the training phase, allowing mice to interact with them for 10 min. On the test day, one familiar object was replaced with a novel object. Considering the visual hypoacuity of aged mice, objects differed in shape to engage multiple senses.

Specifically, the objects used in the NOR test included a transparent polypropylene laboratory vial (height: 12 cm, diameter: 3 cm) and a colored plastic brick (height: 8 cm, base: 3 cm × 3 cm). All objects were cleaned with 70% ethanol between trials to eliminate potential olfactory cues. Mice explored the objects for 10 min. To mitigate bias, object positions were alternated among mice (Hale and Good 2005).

Mouse activity was recorded by a Logitech Brio Ultra HD Webcam 4K 1080 P 60FPS (Logitech Lausanne Switzerland) and analyzed using Biobserve Viewer3 (Biobserve GmbH, Germany).

Object exploration was defined as the nose directed at the object within a 2 cm distance. The arenas were cleaned with 70% ethyl alcohol between sessions to reduce odor cues. Exploration time was measured, and performance was evaluated using the discrimination index (DI) as (*T*_*N*_ − *T*_*F*_)/(*T*_*N*_ + *T*_*F*_) (Lueptow 2017), where *T*_*N*_ is the time spent exploring the novel object and *T*_*F*_ is the time spent exploring the familiar object. Data were excluded if the total exploration time was less than 20 s (Lueptow 2017). An unfiltered DI was also calculated to increase the sample size by including all data regardless of exploration time.

NOR tests were conducted by a single, trained operator across all sessions within each experiment to minimize variability.

### 2.3 Barnes Maze Test

The Barnes Maze test was used to assess spatial learning and spatial reference memory. It was chosen because it provides robust results comparable to those of the more commonly used Morris Water Maze while avoiding the potential stress caused by physical exertion, which is particularly important for geriatric animals (Mizunoya et al. 2004). Test sessions were conducted during the daytime. The arena consisted of a white, circular platform (122 cm diameter) with 20 holes (5 cm diameter) along the perimeter, elevated 30 cm above the ground (Figure S1A). The illumination at the center of the arena was maintained at approximately 1200 lux, in accordance with standard procedures reported in the literature (Blackmore et al. 2022; Illouz et al. 2016; Kesby et al. 2015; Pitts 2018). Bright lights served as negative reinforcement, encouraging the mice to locate the escape box hidden beneath one of the holes. The lamp was positioned at a fixed distance from the testing area to standardize the light intensity, providing uniform light exposure across all animals and sessions. Sessions were recorded with a Logitech Brio Ultra HD Webcam 4K and analyzed using Biobserve Viewer3. The protocol, adapted from Attar et al. (Attar et al. 2013), consisted of three phases: habituation (1 day), acquisition (3 days), and an acquisition probe trial (1 day).

- During habituation, each mouse was placed at the arena's center under a black‐colored beaker. After 10 s, the beaker was removed, allowing the mouse to explore the arena for 3 min. If the mouse failed to enter the escape hole during this time, it was gently guided to the hole, placed inside the escape box, and allowed to remain inside for 1 min. This phase was conducted only once per mouse.
- In the acquisition phase, each mouse performed three trials per day, with a 30‐min inter‐trial interval, for three consecutive days. At the start of each trial, the mouse was placed in the center of the maze under a transparent beaker for 10 s. The beaker was then lifted, and the mouse was allowed to freely explore the maze to locate the escape box. A visual cue (a green wall on the side opposite the escape box) was present in the testing room to facilitate spatial learning. However, since most geriatric mice have impaired vision, this cue likely had little impact on their performance. Mice could rely on alternative strategies, such as tactile, olfactory, and serial‐search cues (Harrison et al. 2006). Each trial lasted a maximum of 120 s. If the mouse did not find the escape box within the time limit, it was gently guided to the box and allowed to remain inside for 1 m. The maze and escape box were cleaned with 70% ethanol between trials to eliminate olfactory cues. The time taken to locate the escape hole (escape latency, EL) was recorded and the mean EL was calculated for each day (mean EL_d1_, mean EL_d2_, mean EL_d3_). Additionally, the difference between the mean performance on day 1 and the mean EL values on day 2 or day 3 (mean EL_d1−d2_ and mean EL_d1−d3_, respectively) was calculated to quantify the improvement in performance over time. Positive values indicate a decrease in EL, reflecting spatial learning (Sharma et al. 2010).
- On the acquisition probe trial day, the escape box was removed. Mice were placed in the arena's center and allowed to explore for 120 s. Two parameters were measured: escape latency (EL_trial_) and time spent in the target quadrant (TSTQ_trial_). EL_trial_ was defined as the time taken by the mouse to locate, for the first time, the escape hole, that is, the location where the escape box was previously positioned. TSTQ_trial_ was defined as the duration spent in the quadrant containing the escape hole, which corresponds to one‐fourth of the entire maze. The reduction of EL_trial_ and/or increased TSTQ_trial_ assess memory retention (Patil et al. 2009). The arena was cleaned with 70% ethanol between each animal to reduce odor cues.

The location of the escape hole remained constant throughout longitudinal tests.

To minimize variability, Barnes maze tests were conducted by a single trained operator across all sessions within each experiment.

### 2.4 Cognitive Frailty Index Computation

The Cognitive Frailty Index (CoFI) was developed to assess cognitive decline across specific measures derived from the Barnes Maze test.

The first step involved converting Barnes Maze test parameters (expressed in s) into severity ratings, aiming to minimize the influence of the animal's physical abilities on the parameters and, consequently, on CoFI. Severity ratings of 0.0 (no alteration), 0.5 (intermediate severity), or 1.0 (altered parameter) were assigned based on predefined rules and cutoff points (Table S2) established from a reference population aged 21 to 27 months.

- EL_d3_ and EL_trial_ Scores: mean EL_d3_ and EL_trial_ values below tertile‐based cutoffs (approximated to 40 s for EL_d3_ and 35 s for EL_trial_) were assigned a score of 0.0, while the maximum score of 1.0 was given to values of 120 s. Values between these cutoffs were scored as 0.5 (Figure S1B,C).
- EL_d1−d3_ Score: animals consistently demonstrating mean EL_d3_ values < 40 s or showing improved or stable performance during the training phase (mean EL_d1−d3_ ≥ 0 s) were assigned a score of 0.0. Conversely, animals with worsened performance (mean EL_d1−d3_ < 0 s) or those never finding the escape hole (120 s on both days 1 and 3) received a frailty value of 1.0 (Figure S1D).
- TSTQ_trial_ Score: tertile‐based cutoff points of TSTQ_trial_ were applied specifically to animals failing to find the escape hole. Animals with an EL_trial_ values of < 120 s received a frailty value of 0.0 for TSTQ_trial_ (Figure S1E). For EL_trial_ values of 120 s, a TSTQ_trial_ values of 0 s received a frailty value of 1.0, while values exceeding the cutoff point of 40 s (approximated from the second tertile of 42.2 s) were scored as 0.0. Values falling between these cutoffs were scored as 0.5 (Figure S1E').

The CoFI was computed as the arithmetic mean of the individual scores derived from EL_d1−d3_, EL_trial_, and TSTQ_trial_. CoFI scores range from 0.0 (indicating highest cognitive functionality) to 1.0 (lowest cognitive functionality). The Cognitive Health Score (CoHS) was computed as the complement of CoFI (1‐CoFI), representing a complementary measure of cognitive health.

### 2.5 Functional Phenotyping of Mice

Physical Function Score (PFS) and Clinical Frailty Index (CFI) in mice were measured as previously described (Marcozzi et al. 2023a). All frailty measurements were performed within the Geriatric Mouse Clinic of IRCCS INRCA in a dedicated area.

Briefly, the PFS comprised five criteria: body size, strength, endurance, speed, and activity. Each criterion was assessed through multiple measurements to ensure testing reliability:

- Body Size Score: composite score reflecting the body condition of the mice that includes current body weight and body length;
- Strength Score: composite score reflecting the forelimb grip strength of the mice measured via various tests (i.e., grip strength meter, home cage lift, and gripping weights lift test);
- Endurance Score: composite score reflecting the endurance capacity of the mice that included treadmill performance, time on a rotarod, and normalized weight‐lifting abilities;
- Speed Score: composite score reflecting four different measurements of speed that included locomotor activity in open field tests, rotarod maximum speed, and stride length analysis;
- Activity Score: composite score related to the dynamic behavior of the mice during a locomotor activity test (i.e., total distance traveled and time spent moving).

The PFS was computed as the average of these scores. The result is a score ranging from 1 (highest functionality) to 0 (lowest functionality). To minimize variability, each test was consistently performed by the same trained operator across all sessions, within each experiment. Table S3 presents the means and SD of the five individual scores as well as the comprehensive PFS for each age category.

The CFI score for each mouse was calculated using the previously published checklist and method (Whitehead et al. 2014). This value ranges from 0 (optimal health) to 1. CFI was also converted to its complementary (1‐CFI) renamed Clinical Health Score (CHS).

CFI assessments were consistently performed by the same trained operator for all sessions within each experiment to ensure consistency and minimize variability.

### 2.6 Adjusted Cognitive Frailty Index Computation

To further minimize the impact of physical performance on the CoFI, we identified the tertiles of PFS in a population of animals aged 21–27 months and categorized the study cohort into three PFS classes based on these PFS tertiles (Table S4).

New tertile‐based cutoff points were then defined for mean EL_d3_, mean EL_d1−d3_, EL_trial_, and TSTQ_trial_ within each PFS class, and parameters were converted into severity rates as follows:

- EL_trial_ Scores: mean EL_trial_ values below defined tertile‐based cutoffs (35 s for high PFS class; 35 s for medium PFS class; 45 s for low PFS class; Table S4) received a score of 0.0. Cutoff points were different depending on the PFS‐based class to which the animal belonged. A maximum score of 1.0 was assigned to values of 120 s. Values falling between these cutoffs were scored as 0.5.

EL_d1−d3_ Score: animals consistently demonstrating mean EL_d3_ under the specific cutoff point (35 s for PFS class 1; 40 s for PFS class 2; 50 s for PFS class 3; Table S4) or showing improved or stable performance during the training phase (mean EL_d1−d3_ ≥ 0) were assigned a score of 0.0. Conversely, animals with worsened performance (mean EL_d1−d3_ < 0) or those never finding the escape hole (120 s on both days 1 and 3) received a frailty value of 1.0.

- TSTQ_trial_ Score: tertile‐based cutoff points of TSTQ_trial_ were applied specifically for animals failing to find the escape hole. Animals with EL_trial_ values of < 120 s received a frailty value of 0.0 for TSTQ_trial_. For EL_trial_ values of 120 s: TSTQ_trial_ values of 0 s received a frailty value of 1.0, while values exceeding the defined tertile‐based cutoff point (45 s for PFS class 1; 40 s for PFS class 2; 45 s for PFS class 3; Table S4) were scored as 0.0. Values falling between these cutoffs were scored as 0.5.

The new score, the adjusted CoFI (adj‐CoFI) was computed as the arithmetic mean of the individual scores derived from EL_d1−d3_, EL_trial_, and TSTQ_trial_. Adj‐CoFI scores range from 0.0 (indicating highest cognitive functionality) to 1.0 (lowest cognitive functionality).

### 2.7 Operator Training and Inter‐Rater Reliability

Details on operator training and inter‐rater reliability are provided in Appendix S1.

### 2.8 Statistical Analysis

Differential survival patterns were estimated by Kaplan–Meier with Log‐Rank test and by Cox regression, accounting for potential confounding variables (age at inclusion and sex).

For longitudinal data analysis, a generalized linear mixed model (GLMM) was used to account for repeated measures on the same subjects and the progressive reduction in sample size over time. The data at each time point include all mice alive at that specific time point. The identifiers of each mouse, sex, and age were included in the model. Target distribution and relationship with the model were estimated on minimal Akaike and Bayesian Information Criteria. Residual methods and robust estimation were used as post‐estimation settings.

The Fisher's exact test was used to evaluate the association between categorical variables, while Spearman's rank correlation coefficient was used to examine the correlation between continuous variables.

Linear mixed model (LMM) analysis was employed to assess the association between CoFI, PFS, and CFI accounting for chronological age. Random intercepts were included in the LMM to account for the repeated measures design of the study, with individual animals treated as random effects to capture intra‐subject variability over time. Target distribution and relationship with the model were estimated on minimal Akaike and Bayesian Information Criteria.

To evaluate the association between frailty and mortality, the study cohort was divided into three subgroups based on frailty assessment: cognitively frail only, physically frail only, and frail in both domains. A GLMM with a binomial distribution and logit link function was applied to analyze the association between frailty categorization, based on CoFI, PFS, or their combination, and mortality within one month. Mouse identifier was incorporated as a random effect to account for inter‐individual variability. Sex and age were included in the model.

Results were considered significant for *p*‐values < 0.05. All the analyses were performed using IBM SPSS Statistics version 29.0.

The influence of test repetition was analyzed by comparing the performance of 27‐month‐old animals undergoing the test for the first time with the performance of animals of the same age that have repeated the test for the second or third time, with a three‐month interval between sessions. Results were presented as mean ± SD, and the *p*‐value was determined by one‐way ANOVA followed by Bonferroni post hoc analyses using GraphPad Prism software (version 7.0, San Diego, CA, USA).

## 3 Results

### 3.1 Study Population and Survival Curves

The characteristics of the study population, including age and sex distribution, are summarized in Table 1. The survival curves of the study population are reported in Figure S2. The mean survival age estimated with Kaplan–Meier was 30.0 ± 0.2 months (males: 28.9 ± 0.3 months; females: 30.3 ± 0.3 months), with the first and last mouse dying at 23.1 and 40.2 months, respectively.

**TABLE 1:** Description of the study population.

Total population	
*n*	424
Male	205
Female	219
*n* recorded deaths (*n* censored)	368 (56)
Male	176 (29)
Female	192 (27)
Age at enrollment (months)	23.65 ± 1.31
Male	23.68 ± 1.32
Female	23.64 ± 1.30
Age at death^a^ (months)	29.99 ± 0.18
Male	28.93 ± 0.25
Female	30.33 ± 0.25

^a^ The age at death was estimated with Kaplan–Meier.

### 3.2 Analysis of Age‐Related Trends in NOR Parameters

Our initial focus was on identifying the performance parameters most sensitive to age‐related cognitive variations. To assess longitudinal changes in cognitive behavior associated with aging, all mice were subjected to the NOR and Barnes Maze test every three months until their natural death. The overall number of recorded phenotypes at each age category is listed in Table S5.

For the NOR test, DI was computed as a measure of the recognition memory of the animal. Data showed no discernible association between age and the time spent exploring the novel object (Figure S3A and Table S6). To avoid the loss of animals due to the filtration of DI based on the minimum standard (Table S6), unfiltered DI was also used; however, the result remained substantially unchanged (Figure S3B and Table S6).

To investigate the potential test repetition effect on NOR performance, we compared the DI of 27‐month‐old mice that underwent the NOR test for the first time with that of animals of the same age undergoing the NOR test for the second or third time. Statistical analysis revealed no significant differences in DI values between these groups, indicating that mice did not retain memory of the previous testing sessions after a three‐month interval (Figure S3C).

### 3.3 Analysis of Age‐Related Trends in Barnes Maze Parameters

**FIGURE 1:**
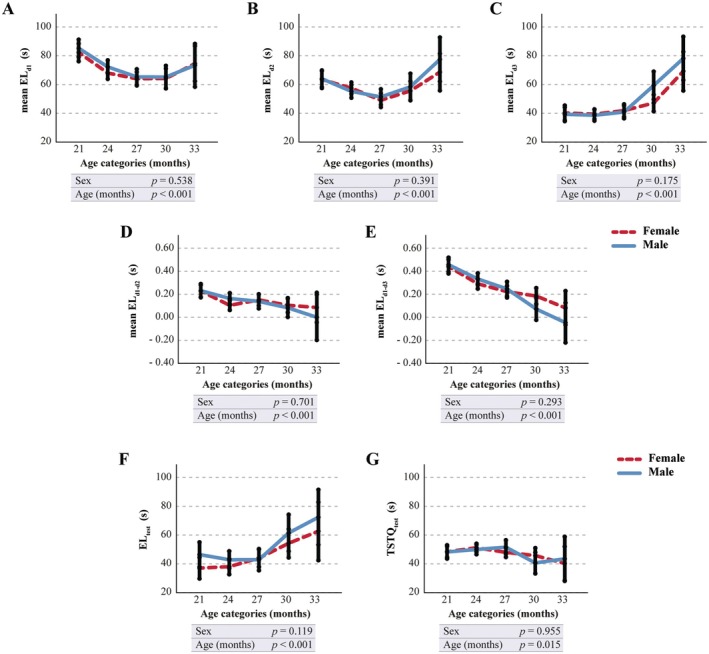
Quantitative assessment of performance parameters derived from the Barnes Maze test. C57BL/6J mice (*n* = 424) were monitored every three months from inclusion up to natural death. (A–E) Graphs showing the trend with advancing age of the mean latency to enter the escape hole at day 1 (A), day 2 (B), and day 3 (C) of the acquisition phase, both in male (blue solid line) and female (red dashed line) mice. Subtraction of mean EL values on day 2 or day 3 from the mean performance on day 1 is also reported (D and E), both in male (blue solid line) and female (red dashed line) mice. Time is expressed in age categories. Values are expressed in s and reported as the mean estimates (95% CI) obtained by generalized linear mixed model analysis for longitudinal data, using sex, cohort, and age (months) as fixed factors. Tests of fixed effects parameters (sex and age) are reported inside the figure. (F–G) Graphs showing the trend with advancing age of the latency to enter the escape hole (F) and the time spent in the target quadrant (G) during the acquisition probe trial, both in male (blue solid line) and female (red dashed line) mice. Time is expressed in age categories. Values are expressed in s and reported as the mean estimates (95% CI) obtained by generalized linear mixed model analysis for longitudinal data using sex, cohort, and age (months) as fixed factors. Test of fixed effects parameters (sex and age) are reported inside the figure. Mean EL_d1_: escape latency calculated as the mean of the three repetitions on day 1; mean EL_d2_: escape latency calculated as the mean of the three repetitions on day 2; mean EL_d3_: escape latency calculated as the mean of the three repetitions on day 3; mean EL_d1−d2_: mean escape latency on day 1 minus mean escape latency on day 2; mean EL_d1−d3_: mean escape latency on day 1 minus mean escape latency on day 3; EL_trial_: escape latency during the acquisition probe trial; TSTQ_trial_: time spent in the target quadrant during the acquisition probe trial. Age category 21: 21–23 months of age; age category 24: 24–26 months of age; age category 27: 27–29 months of age; age category 30: 30–32 months of age; age category 33: 33–36 months of age.

For the Barnes Maze test, analysis of age‐related trends in EL indicated that mean EL on day 3 (mean EL_d3_) had the strongest correlation with age (Figure 1A–C and Table S6). Furthermore, computing the differences of mean EL values on day 2 (mean EL_d2_) or day 3 (mean EL_d3_) from day 1 (mean EL_d1_) consistently revealed the strongest correlation with aging on the third day of the acquisition phase (mean EL_d1−d3_) (Figure 1D,E and Table S6). Notably, EL_d1−d3_ values consistently exceed those of EL_d1‐d2_, confirming that learning improves with increasing days and trials. Additionally, the data suggest a significant age‐related change in the EL during the acquisition probe trial (EL_trial_) and in the time spent in the target quadrant (TSTQ_trial_) (Figure 1F,G and Table S6).

### 3.4 Influence of Test Repetition on Barnes Maze Parameters

To investigate the hypothesis that animals retain memory of the test across sessions, we compared the performance parameters of 27‐month‐old animals undergoing the test for the first time with those of animals of the same age repeating the test for the second or third time, with a three‐month interval between sessions. It is worth mentioning that the analysis was conducted on 27‐month‐old animals as this age falls within a range where the variables are minimally influenced by age, and additionally, a sufficiently robust sample size for the three distinct groups was available, ensuring reliable data.

The results indicate that, at equivalent ages, 27‐month‐old mice significantly improved their performances (revealed as a significant reduction of mean EL_d1_ and EL_d2_) as the number of repetitions increased, suggesting a carryover effect and a bias due to test repetition (Figure S4A–C). The variable mean EL_d1–d3_ was influenced consequently (Figure S4D). Conversely, the mean EL_day3_ and data acquired on the test day (EL_trial_ and TSTQ_trial_) remain unaffected by the repetition of the test (Figure S4E,F).

### 3.5 EL_d3_ , EL_trial_ , TSTQ_trial_ , and EL_d1_ _−d3_ Scores

Parameters like EL (expressed in s) are highly dependent on the physical condition (i.e., activity and velocity) of the animal. To reduce the influence of the physical health of the mice on the measured parameters, we transformed the parameters that were more closely associated with age into severity rates. Specifically, each criterion was graded with a severity rate of 0.0, 0.5, or 1.0 as reported in M&M, where higher values indicate an increased alteration of the parameter.

The prevalence of 0.0, 0.5, or 1.0 score assigned to each performance parameter at each age category showed that the percentage of mice over 27 months of age scored as 1.0 or 0.5 increased exponentially with age (Figure S5).

It is worth noticing that, when using the scores instead of numerical data to investigate the influence of test repetition on performances, mice categorized as 0.5 based on EL_d3_ tend to be more prevalent in the group undergoing the Barnes Maze test for the first time compared to animals with prior test experience (Figure S6A). In contrast, the frailty categorization rule for the computation of the EL_d1−d3_ score helps mitigate the repetition effect, with approximately 85% of 27‐month‐old animals classified as normal regardless of test repetition (Figure S6B). Importantly, there is no influence of repetition on the frailty classification based on EL_trial_ score and TSTQ_trial_ score (Figure S6C,D).

### 3.6 Cognitive Frailty Index (CoFI)

**FIGURE 2:**
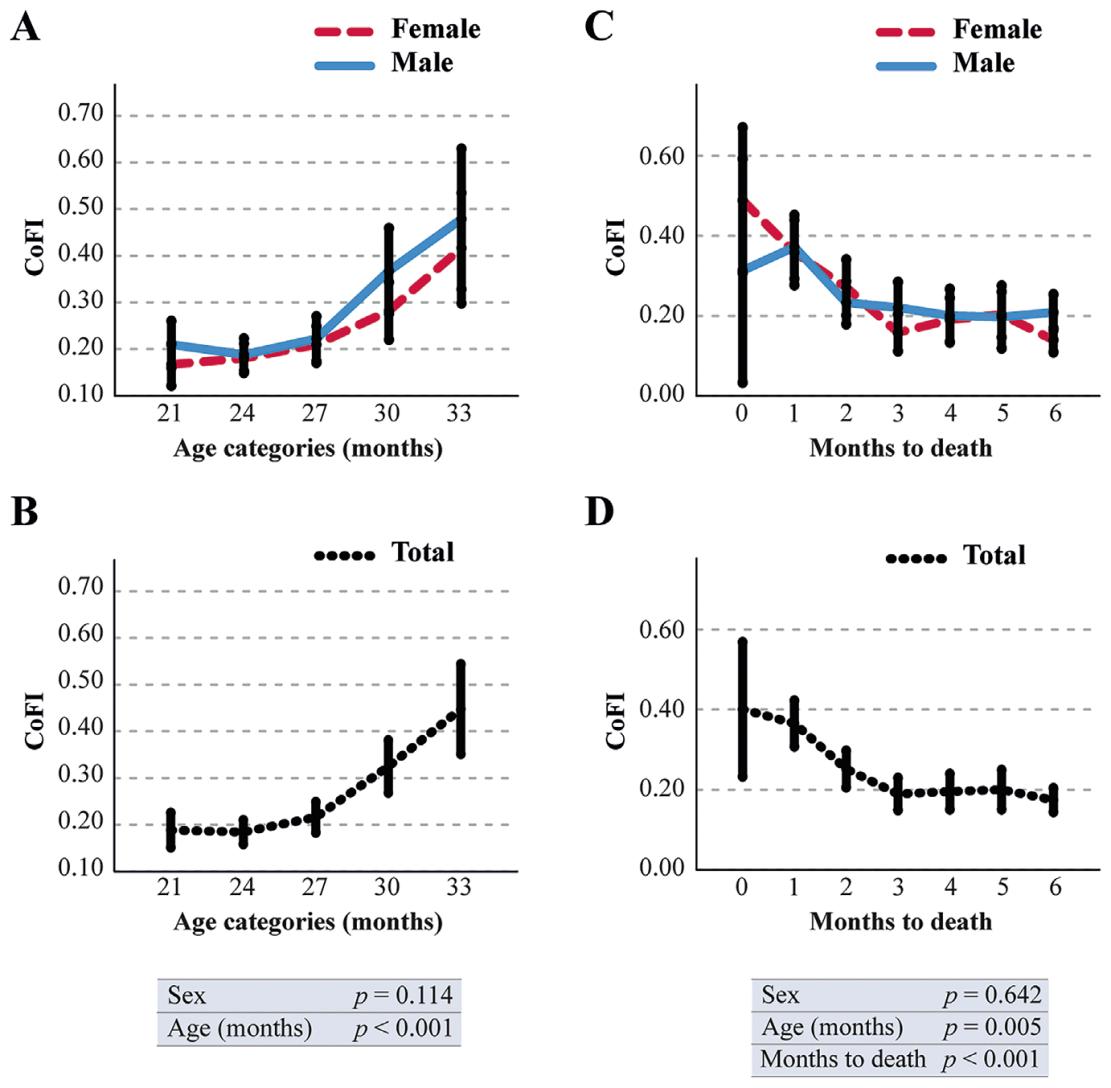
Association of the Cognitive Frailty Index (CoFI) with aging and mortality. C57BL/6J (*n* = 424) mice were monitored every three months from inclusion up to death. Data of CoFI are reported as a function of age categories (A, B) or months to death (C, D). Time is expressed in age categories (A, C) or as months to death (B, D), respectively. Values are reported as the mean estimates (95% CI) obtained by generalized linear mixed model analysis for longitudinal data using sex, cohort, and age (months) as fixed factors. Tests of fixed effects parameters (sex, age, or months to death) are reported inside the figure. Data from male mice (blue solid line), female mice (red dashed line), or whole population (black dotted line) are presented.

For the computation of CoFI, we selected the scores that are both associated with advancing age and unaffected by test repetition, specifically the individual scores derived from EL_d1−d3_, EL_trial_, and TSTQ_trial_ (Figure S7). The CoFI was derived as the arithmetic mean of the frailty values assigned to these parameters. The data in Figure 2A,C demonstrate that the CoFI significantly correlates with advancing age. More precisely, it exhibits a slight and gradual increase to 27 months of age, succeeded by a rapid acceleration thereafter. This variable remained unaffected by sex in its temporal progression. Moreover, CoFI is significantly associated with mortality: graphs in Figure 2B,D depict a rapid increase in CoFI as mortality approaches. This association was independent of sex and remained statistically significant even when chronological age was included in the analysis.

### 3.7 Validation Model: Long Lived Versus Short Lived Animals

**FIGURE 3:**
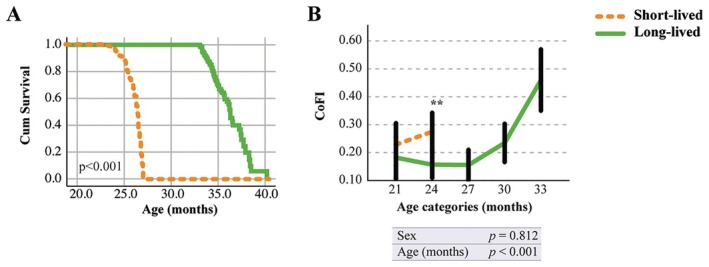
Cognitive Frailty Index (CoFI) validation. (A) Cox regression survival curve of short‐lived (orange dashed line, *n* = 164) versus long‐lived (green solid line, *n* = 65) C57BL/6J mice adjusted for sex, age at inclusion, and cohort. Mortality occurred when mice died naturally or were euthanized for aging‐associated diseases. The analysis demonstrates a significant difference in survival probability between the two groups (*p* < 0.001). (B) CoFI was monitored every three months from inclusion up to death in short‐lived and long‐lived groups. Time is expressed in age categories. Values are reported as the mean estimates (95% CI) obtained by generalized linear mixed model analysis for longitudinal data using group, sex, and age (months) as fixed factors. Tests of fixed effects parameters (sex and age) are reported inside the figure. **p < 0.01.

To assess the discriminative efficacy of CoFI across different treatments/conditions, we next subdivided our initial cohort of mice into two categories based on the animal's age at death: a short‐lived group (mice that died within 27 months of age) and a long‐lived group (mice that survived beyond 33 months of age). The mean age at death was 25.1 ± 1.4 and 34.9 ± 1.8 months for short‐lived and long‐lived mice, respectively (Table S7). Reduced lifespan in the short‐lived group compared to the long‐lived group was confirmed by the Cox regression survival curve (Figure 3A). To investigate whether the differences in lifespan were associated with a significant decline in cognitive performances, the CoFI was analyzed in the two lifespan categories. Notably, the trend shown in Figure 3B revealed a significant difference in CoFI between the short‐lived and long‐lived groups. This means that mice with higher CoFI levels exhibit a shorter lifespan.

### 3.8 Relationship Between CoFI and PFS

Recently, we developed an alternative method for assessing frailty in aged mice, in addition to the widely validated CFI: the PFS (Marcozzi et al. 2023a). This continuous variable, proposed as an effective measure of physical health in mice, was correlated with advancing age and exhibited a significant association with mortality (Marcozzi et al. 2023a).

**FIGURE 4:**
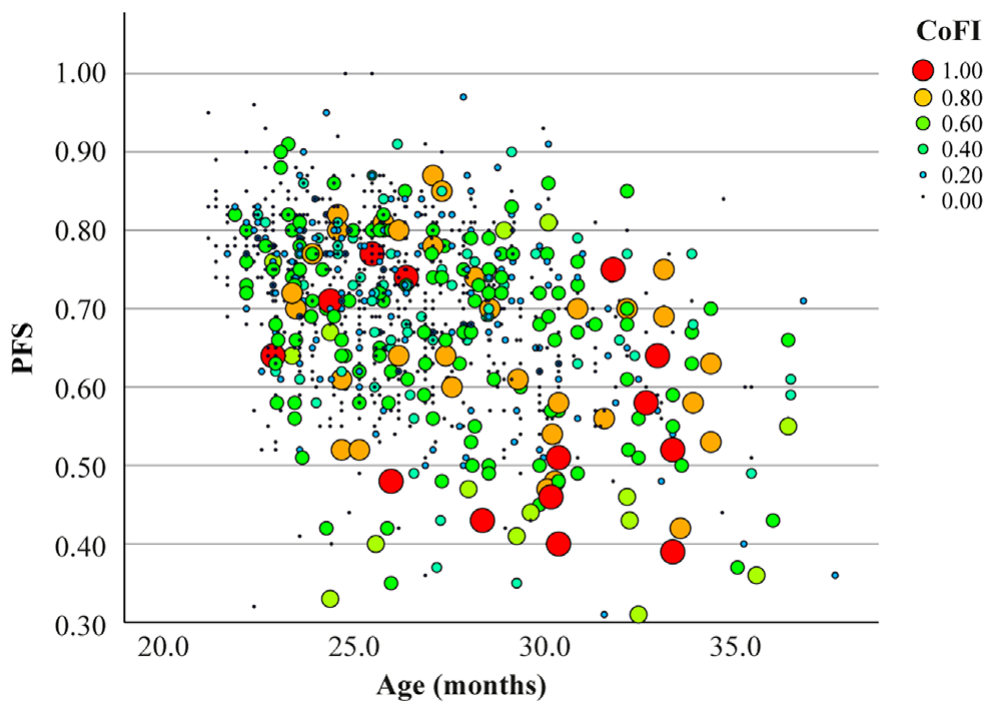
Graphical representation of the relationship between CoFI, PFS, and age. The bubble plot shows the distribution of PFS as a function of age, with the size and color of the dots representing CoFI values. Larger and red/orange dots indicate higher CoFI values, while smaller and green dots represent lower CoFI values. CoFI: Cognitive Frailty Index; PFS: Physical Function.

To determine if the newly created CoFI is influenced by physical health, its association with PFS was analyzed. Spearman's correlation revealed a significant negative correlation (*ρ* = −0.233, *p* < 0.001), suggesting that lower physical function is associated with higher cognitive deterioration (Figure 4). However, both CoFI and PFS are significantly associated with age (Figure 2 and Marcozzi et al. 2023a, respectively). To account for the potential confounding effect of age and the longitudinal design, we performed an LMM analysis (Table 2). This analysis showed that PFS was not significantly associated with CoFI after adjusting for age. Moreover, a significant interaction between PFS and age was observed, suggesting that the influence of PFS on CoFI may vary depending on the age of the animals. Overall, these results suggest that CoFI is not directly influenced by PFS and that the observed correlation may be largely mediated by their shared dependence on age.

**TABLE 2:** Association between CoFI and PFS or CFI and interaction with age.

Model	Outcome	Predictor	Main effect		Interaction with age	
			Estimate	*p* value	Estimate	*p* value
1	CoFI	PFS	0.638	0.241	−0.042	0.039
2	CoFI	CFI	−0.508	0.510	0.044	0.121

*Note:* Linear Mixed Model analysis was performed including the age (months) in the model. The outcome refers to the dependent variable being measured, while the predictor represents the independent variable or factor under investigation. The main effect columns present the estimated value and *p* value for the relationship between the predictor and the outcome. The estimate and *p* value for the interaction effect between the predictor and age on the outcome are also reported.

To further investigate the impact of physical health on cognitive function, we next examined the association between physical and cognitive frailty. Firstly, the CoFI was converted into a categorical variable by identifying a cutoff value to define the onset of frailty. Based on the 95th percentile, the cutoff was established at 0.83. The bar graph depicted in Figure S8 illustrates a gradual increase in the percentage of mice classified as frail with advancing age. Using the previously established frailty cutoff for PFS (0.55), we explored the overlap between animals classified as frail by the two different indices. Data in Table S8 indicated discrepancies, with some animals classified as frail by PFS that are not frail for CoFI, and vice versa, and only a small number of animals that are classified as frail for both indices. Statistical analysis revealed associations in the 24‐ and 27‐month age categories between the two categorical variables, whereas no significant association is observed at 21‐, 30‐, and 33‐month age categories.

Further analysis among the three subpopulations—cognitively frail, physically frail, and frail in both domains—revealed a strong association with mortality within one month. Specifically, while being frail in either the cognitive or physical domain was associated with an increased risk of mortality, the combination of frailty in both domains was a stronger predictor of mortality (Table S9).

### 3.9 CoFI Adjustment for Physical Function

To further reduce the confounding effect of physical performance on the CoFI, we create an adjusted CoFI (adj‐CoFI) that takes into account the PFS of animals as well. After adjustment, the new index remained significantly correlated with advancing age (Figure S9A,C) and mortality (Figure S9B,D). When adj‐CoFI was converted into a categorical variable using the cutoff of 0.83, the relationship with PFS was slightly reduced with respect to CoFI, with occasional associations noted only in the 21‐month age category (Table S10). The analysis of frailty‐related mortality risk within 1 month, using frailty defined by adj‐CoFI, PFS, or both, yielded results consistent with those observed using the CoFI (Table S9).

### 3.10 CoFI, CFI, And PFS

To assess aging dynamics across different health domains, we compared physical (PFS), clinical (CFI), and cognitive (CoFI) indices. A simple bubble plot including all data suggests that mice with higher CoFI values tend to cluster at intermediate CFI values and lower PFS scores, indicating that animals with pronounced cognitive frailty often exhibit moderate overall clinical frailty alongside reduced physical function (Figure S10). However, these correlations might be influenced by age, as our previous studies (Marcozzi et al. 2023a) highlighted a strong association between CFI and age. To investigate the relationships between the three indices, while accounting for the potential confounding effect of age, we employed LMM analysis (Table 2). As for PFS and CoFI, analysis of the relationship between CoFI and CFI revealed no significant main effect of CFI on CoFI, nor was the interaction between CFI and age significant. Overall, these findings suggest that while some correlations exist among CFI, CoFI, and PFS, these relationships are complex and likely influenced by age‐related factors.

**FIGURE 5:**
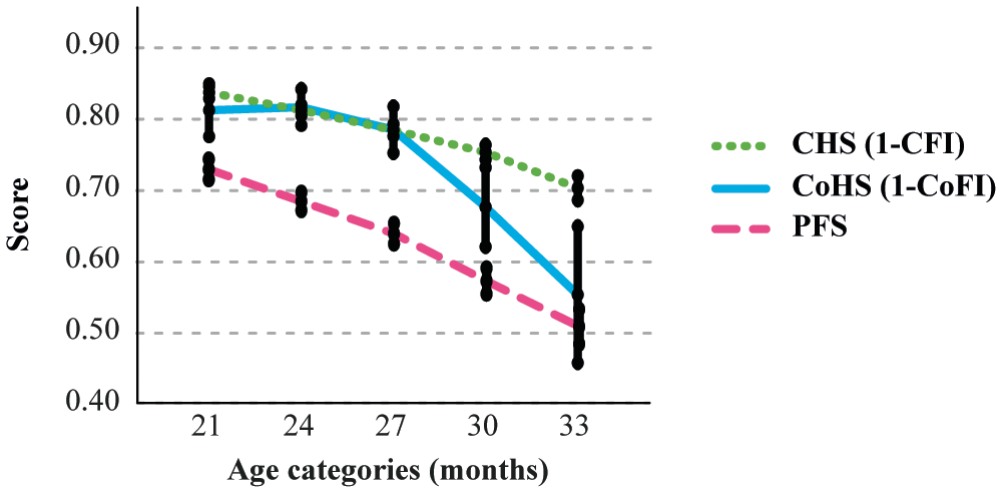
Trend of the three aging domains in mice. The graph overlays the trajectories of clinical (CHS, green dotted line), physical (PFS, purple dashed line), and cognitive (CoHS, blue solid line) aging domains. While physical and clinical health decline steadily over time, cognitive deterioration manifests later in aging but then accelerates more rapidly than other domains. Time is expressed in age categories. Values are reported as the mean estimates (95% CI) obtained by generalized linear mixed model analysis for longitudinal data using group, sex, and age (months) as fixed factors. CFI was converted to its complementary (1‐CFI) renamed Clinical Health Score (CHS). CoFI was also converted to its complementary (1‐CoFI) renamed Cognitive Health Score (CoHS).

To gain a clearer picture of the aging dynamics across these health domains, we transformed CFI and CoFI into their complementary measures—CHS and CoHS, respectively—enabling a direct comparison with PFS. Overlaying these trends confirmed that all three indices follow age‐correlated trajectories but with distinct patterns: CFI and PFS exhibited gradual declines over time, whereas CoFI showed a more pronounced decline after 27 months compared to the other measures (Figure 5). This divergence suggests differing patterns in aging dynamics among the assessed domains. A noteworthy case illustrating divergent physical and cognitive trajectories is presented in Video 1.

## 4 Discussion

Our new index of cognitive frailty, termed as CoFI, has been developed to monitor longitudinal alterations in cognitive function, which could be useful during the study of interventions aimed at counteracting aging and age‐related pathologies. For the development of this index, we collected data from two widely used cognitive tests, NOR test and Barnes Maze test.

The NOR test, based on the evaluation of animals' innate exploratory behavior, is a widely used tool for studying physiological memory function and disease‐related cognitive impairment (Ennaceur and Delacour 1988). However, while effective for comparing well‐defined age groups (e.g., young vs. middle‐aged) (Fahlstrom et al. 2011; Roda et al. 2023; Traschutz et al. 2018), its utility for tracking cognitive changes within already aged populations is unknown. In contrast to previous studies, our longitudinal approach followed aged animals as they progressed into advanced geriatric stages, rather than comparing younger and older cohorts. Our findings revealed that the NOR test failed to detect any significant memory consolidation impairment in the 21–36 months age range.

This apparent discrepancy with the existing literature might be attributed to the experimental set‐up and the advanced age of the animals at enrollment. This shift in focus may have altered the sensitivity of the NOR test. Indeed, in our cohort, DI values were always consistently lower than 0.2, indicating a slight side preference and an altered exploratory behavior already at the time of enrollment (21‐month‐old cohort). To rule out potential confounding effects of repeated longitudinal NOR sessions (conducted every three months), we compared the DI of animals of the same chronological age (27 months) tested for the first, second, or third time. This analysis revealed no significant differences between groups (Figure S3), suggesting that mice do not retain memory of prior testing sessions over a three‐month interval. These observations underscore that, while the NOR test is a robust tool for detecting cognitive decline across distinct age cohorts, its discriminatory power might be lost when applied in geriatric cohorts, making it less effective at identifying subtle cognitive decline in older animals. Therefore, the choice of experimental design is crucial when using the NOR test to study late‐life cognitive trajectories, emphasizing the need for careful interpretation based on the specific context.

The Barnes Maze test is widely considered the gold standard for evaluating spatial learning and spatial reference memory evaluation in aged rodents (Barrett et al. 2009; Harrison et al. 2009; Kennard and Woodruff‐Pak 2011; Morel et al. 2015). In our study population, we analyzed the mean escape latency (EL) each day of the acquisition phase. Specifically, the mean EL_d1_ (i.e., the average of the three values obtained during the first day of the trial) provides insights into the rapid acquisition of spatial information, which might be considered a component of working memory (Rosenfeld and Ferguson 2014). Notably, in our experimental setup, working memory seems to be unaffected by aging, as indicated by the absence of age‐related effects in this Barnes‐derived parameter in the tested cohort. Conversely, the prominent age‐related effect observed in the mean EL_d3_ indicates an impairment with aging in retrieving information from the preceding days of the acquisition phase. This strong correlation with age is further emphasized by the decline of the mean EL_d1–d3_, a parameter reflecting the performance improvement by the animal during the acquisition phase. The tendency of this parameter to approach 0 with increasing age, indicating an age‐related inability to improve performance over successive days, underscores impaired recall and spatial learning functions, particularly in animals older than 27 months (Gawel et al. 2019; Sharma et al. 2010). Age‐related trends were also observed during the acquisition probe trial in the latency (EL_trial_) and time spent in the correct zone (TSTQ_trial_): these parameters reflect spatial memory retention (Gawel et al. 2019; Patil et al. 2009). Overall, the examination of Barnes Maze test parameters revealed a significant decline in spatial learning and spatial memory functions.

**VIDEO 1:** A noteworthy case illustrating divergent physical and cognitive trajectories is presented in this video. Despite exhibiting compromised physical function, including motor coordination and vestibular issues, the mouse performs the cognitive task (Barnes test, Probe day), demonstrating preserved memory and executive function. Video content can be viewed at https://onlinelibrary.wiley.com/doi/10.1111/acel.70056

It is important to highlight that, given the advanced age of the mice, the performance on the tasks required by the Barnes Maze test may have been influenced by their physical health. To account for this, the four parameters that were most strongly associated with increasing age (mean EL_d3_, mean EL_d1−d3_, EL_trial_, and TSTQ_trial_) were transformed into severity scores. These new variables (EL_d3_ score, EL_d1−d3_ score, EL_trial_ score, and TSTQ_trial_ score) were calculated using broad cutoff points. By using severity scores instead of raw values for index computation, it is possible to minimize the impact of the animal's physical capabilities on the CoFI score. Parameters like EL and TSTQ (expressed in s) are heavily influenced by the animal's physical health: a physically frail animal will inevitably have a longer EL compared to physically fit animals. Assigning a severity grade of 0.0 to an animal that finds the escape hole within a wide time window allows one to classify a physically frail but cognitively intact animal as cognitively healthy, similar to a physically robust animal.

Another consideration is that previous studies suggest repeated exposure to cognitive tests may influence mice performance due to carryover effects from prior experiences (Cnops et al. 2022). In accordance, the comparison between 27‐month‐old animals taking the Barnes Maze test for the first time and those of the same age with prior experience highlights an interesting dynamic. While mice with previous exposure to the test showed more optimal EL_d3_ score categorization, the EL_d1−d3_ score, EL_trial_, and TSTQ_trial_ scores were unaffected by test repetition (Figure S6). This suggests that these parameters are robust indicators of cognitive impairment, without bias from previous exposure to the test.

Based on these findings, we computed CoFI considering only the scores derived from the Barnes Maze test parameters that associate better with age and that were unaffected by test repetition, to create an intuitive continuous value ranging from 0.0 (highest cognitive functionality) to 1.0 (lowest cognitive functionality). Specifically, the CoFI was derived from three key parameters: EL_d1−d3_, which reflects recall and spatial learning functions, and EL_trial_ and TSTQ_trial_, which assess spatial memory retention. While Barnes Maze parameters are traditionally analyzed separately to investigate specific cognitive domains, such as memory or learning (Barreto et al. 2010; Ederer et al. 2022; Shoji et al. 2016; Yanai and Endo 2021; Yang et al. 2019), we integrated them into a single index. Learning refers to the process of acquiring new information, while memory refers to the ability to retain and recall that information (Kandel et al. 2000). Understanding both aspects is crucial for capturing a comprehensive profile of cognitive function. This approach enables the CoFI to serve as a comprehensive indicator of cognitive performance, capturing two distinct cognitive domains: spatial learning and spatial memory. The newly generated CoFI significantly increased with advancing age and was strongly associated with overall mortality, with no significant sex‐related differences. Its importance as a cognitive assessment tool in aging research is further supported by the validation model (long‐lived vs short‐lived mice), which clearly demonstrated the effectiveness of CoFI in discerning between different experimental conditions.

To confirm the effectiveness of our strategies in minimizing the influence of physical functionality on the CoFI (i.e., severity rating of Barnes‐derived parameters), we analyzed the relationship between CoFI and PFS. Initially, Spearman's correlation analysis showed a moderate but significant relationship between CoFI and PFS. However, this correlation likely reflects the shared influence of age. After accounting for the effect of age, the association between CoFI and PFS became weak and not statistically significant. This finding reinforces the reliability of CoFI as an indicator of cognitive function independent of the animals' physical health. This is supported also by the discrepancies in the classification of frail mice according to CoFI compared to PFS, which is a scoring method that resumes the five criteria included in Fried's frailty phenotype (Marcozzi et al. 2023a). The observed associations between CoFI and PFS are minimal, indicating that while both indices are related to mortality risk, they capture distinct dimensions of frailty. This is further supported by their enhanced predictive power when used together to assess short‐term mortality.

As further evidence, we tested an adjusted version of the CoFI that accounts for the PFS in its computation. This adjusted CoFI (adj‐CoFI) remained significantly associated with age and mortality. However, despite the adjustment, the classification of cognitively frail mice was only minimally affected compared to that of the original CoFI. Given that the CoFI is easier to evaluate than the adj‐CoFI, as it does not require the assessment of all parameters included in the PFS, we propose that it remains a valuable and practical tool for assessing cognitive frailty.

An advantage in assessing cognitive decline as a continuous variable lies in the possibility of comparing and integrating information from this parameter with other continuous scores, such as the PFS (Marcozzi et al. 2023a) and the CFI (Whitehead et al. 2014). Here, we highlight a key independence between CFI and CoFI: when age is considered in the statistical model. Despite their independence, these indices are complementary: their integration is essential for obtaining a complete picture of aging, particularly in translational studies. In humans, the Frailty Index generally includes clinical, cognitive, and mental health parameters. In rodents, however, the CFI traditionally excludes cognitive dimensions, limiting its ability to reflect the multifaceted nature of aging (Marcozzi et al. 2023b). By integrating CoFI with CFI, we address this gap, aligning rodent measures more closely with human indices and enhancing the translational relevance of preclinical studies. Moreover, comparing the trajectories of age‐related health across the three domains—physical, clinical, and cognitive—revealed distinct patterns. While physical and clinical health decline steadily over time, cognitive deterioration manifests later in aging but with a more rapid progression. This study stands out as one of the few, if not the only, attempts to comprehensively describe aging in a large cohort of geriatric mice by concurrently examining its clinical, physical, and cognitive aspects. In humans, there is ongoing debate regarding the chronological sequence of age‐related changes. Some research suggests that cognitive aging may precede other symptoms, while other studies indicate it may follow (Boyle et al. 2010; Han et al. 2023; Li et al. 2023; Xue et al. 2021). By providing a more nuanced understanding of how physical, clinical, and cognitive health interact during aging, this framework lays the foundation for more effective preclinical research and therapeutic interventions aimed at mitigating age‐related frailty.

In conclusion, the CoFI stands out as a highly valuable tool, providing a straightforward and intuitive representation of cognitive impairment associated with aging in C57BL/6J mice. Given the validity and widespread use of the Barnes Maze test across various strains and mouse models, there is no reason to suspect that this would be different for other mouse strains. The ability to longitudinally track cognitive decline in old mice using the CoFI has the potential to advance our understanding of the neurobiological origins of age‐related cognitive impairments and to serve as a useful resource for testing new strategies aimed at mitigating the cognitive decline associated with aging. Additionally, the methodology used to construct the CoFI is adaptable and can evolve to incorporate data from additional cognitive assessment tests, such as Y/T‐maze or Elevated Plus maze tests. This flexibility ensures that the CoFI can evolve into a more composite index, reflecting a broader spectrum of cognitive functions. Furthermore, the possibility to integrate CoFI with other frailty assessment tools, such as CFI and PFS, opens avenues for further studies and applications focused on promoting healthspan across different domains.

## Author Contributions

Conceptualization: M.M.; Data curation: M.M. and S.M.; Formal analysis: M.M. and S.M.; Funding acquisition: M.M., F.L., P.L.J.deK., M.C., F.d'A.d.F.; Investigation: S.M., G.B., M.E.G., G.L., B.B., and A.A.; Resources: F.O.; Methodology: M.M. and M.B.; Project administration: M.M.; Supervision: M.M.; Visualization: S.M.; Writing – original draft: S.M. and M.M.; Writing – review and editing: G.B., M.E.G., G.L., B.B., A.A., M.B., T.C., F.P., R.G., F.L., F.O., P.L.J.deK., M.C., F.d.A.d.F. All authors have read and agreed to the published version of the manuscript.

## Ethics Statement

Authorizations and ethical approval for animal experiments are reported in detail in the section: “Animals and Experimental Design”.

## Conflicts of Interest

The authors declare no conflicts of interest.

## Supporting information


Appendix S1.

Data S1.

## Data Availability

The data that support the findings of this study are available from the corresponding author (M.M.) upon reasonable request.
