# Supplementary material for: A Novel Cognitive Frailty Index for Geriatric Mice
Source: Aging Cell. 2025 May 21;24(7):e70056. doi: 10.1111/acel.70056 (PMC12266745; doi:10.1111/acel.70056)
Supplement: Supplementary file 1 — Appendix S1. [file ACEL-24-e70056-s001.docx]

**A novel Cognitive Frailty Index for Geriatric Mice**

Serena Marcozzi ^1^, Giorgia Bigossi ^1^, Maria Elisa Giuliani ^1^, Giovanni Lai ^1^, Beatrice Bartozzi ^2^, Marta Balietti ^3^, Tiziana Casoli ^3^, Fiorenza Orlando ^4^, Andrea Amoroso ^5^, Robertina Giacconi ^2^, Maurizio Cardelli ^2^, Francesco Piacenza ^2^, Fabrizia Lattanzio ^6^, Fabiola Olivieri ^7,8^, Peter L. J. de Keizer ^9,10^, Fabrizio d’Adda di Fagagna ^11,12^, Marco Malavolta ^1,8^*

**Affiliations**

^1^ Advanced Technology Center for Aging Research and Geriatric Mouse Clinic, IRCCS INRCA, 60121 Ancona, Italy

^2^ Advanced Technology Center for Aging Research, IRCCS INRCA, 60121 Ancona, Italy

^3^ Center for Neurobiology of Aging, IRCCS INRCA, 60121 Ancona, Italy

^4^ Experimental Animal Models for Aging Unit, Scientific Technological Area, IRCCS INRCA, 60015 Falconara Marittima (AN), Italy

^5^ Charles River Laboratories, 23885, Calco, Italy

^6^ Scientific Direction, IRCCS INRCA, 60121 Ancona, Italy

^7^ Advanced Technology Center for Aging Research, IRCCS INRCA, Ancona, Italy

^8^ Department of Clinical and Molecular Sciences, DISCLIMO, Università Politecnica delle Marche, Ancona, Italy

^9^ Center for Molecular Medicine, Division of Laboratories, Pharmacy and Biomedical Genetics, University Medical Center Utrecht, Utrecht, The Netherlands

^10^ Cleara Biotech B.V., Utrecht, The Netherlands^.^

^11^ IFOM ETS - The AIRC Institute of Molecular Oncology, Milan, Italy.

^12^ Institute of Molecular Genetics IGM-CNR "Luigi Luca Cavalli-Sforza", Pavia, Italy

* Correspondence to: Marco Malavolta, Advanced Technology Center for Aging Research, IRCCS INRCA, 60121 Ancona, Italy. **Email:** [m.malavolta@inrca.it](mailto:m.malavolta@inrca.it)

**Supplementary Materials and Methods**

**Humane endpoint criteria**

If signs of distress or health deterioration were observed during the study, a detailed assessment was conducted based on predefined humane endpoint criteria. This evaluation included a series of parameters including body temperature, behavior, body weight, clinical manifestation (e.g. Grimace Score), skin condition, respiration, locomotion, posture, coat condition, eye condition, and visible tumors. Each parameter was scored on a severity scale from 1 to 4; if the total score exceeds the threshold of 15 and the situation is deemed irreversible (monitored for 24-48 hours) despite possible interventions (e.g., facilitating food or water intake, disinfecting wounds, trimming of overgrown teeth), euthanasia was performed in accordance with ethical guidelines. Additionally, a severity score of 4 for certain critical indicators could warrant immediate euthanasia at the discretion of the veterinarian.

**Operator Training and Inter-rater Reliability**

To ensure data consistency and comparability, rigorous training protocols were implemented for all personnel involved data collection.

New team members underwent a structured training program before their involvement in any experiment. This phase consisted of supervised practice sessions during which their data were systematically compared to those performed by an experienced operator. The comparison focused on identifying and reducing variability between the trainee's results and those of the expert. Trainees remained in the training phase until their measurements demonstrated statistical alignment with those of the experienced operator.

By implementing this rigorous training and assessment protocol, we ensured that all operators were proficient and that data across different projects, experiments, and time points were reliable and reproducible.
