# Supplementary material for: A Novel Cognitive Frailty Index for Geriatric Mice
Source: Aging Cell. 2025 May 21;24(7):e70056. doi: 10.1111/acel.70056 (PMC12266745; doi:10.1111/acel.70056)
Supplement: Supplementary file 2 — Data S1. [file ACEL-24-e70056-s002.docx]

**A novel Cognitive Frailty Index for Geriatric Mice**

Serena Marcozzi ^1^, Giorgia Bigossi ^1^, Maria Elisa Giuliani ^1^, Giovanni Lai ^1^, Beatrice Bartozzi ^2^, Marta Balietti ^3^, Tiziana Casoli ^3^, Fiorenza Orlando ^4^, Andrea Amoroso ^5^, Robertina Giacconi ^2^, Maurizio Cardelli ^2^, Francesco Piacenza ^2^, Fabrizia Lattanzio ^6^, Fabiola Olivieri ^7,8^, Peter L. J. de Keizer ^9,10^, Fabrizio d’Adda di Fagagna ^11,12^, Marco Malavolta ^1,8^*

**Affiliations**

^1^ Advanced Technology Center for Aging Research and Geriatric Mouse Clinic, IRCCS INRCA, 60121 Ancona, Italy

^2^ Advanced Technology Center for Aging Research, IRCCS INRCA, 60121 Ancona, Italy

^3^ Center for Neurobiology of Aging, IRCCS INRCA, 60121 Ancona, Italy

^4^ Experimental Animal Models for Aging Unit, Scientific Technological Area, IRCCS INRCA, 60015 Falconara Marittima (AN), Italy

^5^ Charles River Laboratories, 23885, Calco, Italy

^6^ Scientific Direction, IRCCS INRCA, 60121 Ancona, Italy

^7^ Advanced Technology Center for Aging Research, IRCCS INRCA, Ancona, Italy

^8^ Department of Clinical and Molecular Sciences, DISCLIMO, Università Politecnica delle Marche, Ancona, Italy

^9^ Center for Molecular Medicine, Division of Laboratories, Pharmacy and Biomedical Genetics, University Medical Center Utrecht, Utrecht, The Netherlands

^10^ Cleara Biotech B.V., Utrecht, The Netherlands^.^

^11^ IFOM ETS - The AIRC Institute of Molecular Oncology, Milan, Italy.

^12^ Institute of Molecular Genetics IGM-CNR "Luigi Luca Cavalli-Sforza", Pavia, Italy

* Correspondence to: Marco Malavolta, Advanced Technology Center for Aging Research, IRCCS INRCA, 60121 Ancona, Italy. **Email:** [m.malavolta@inrca.it](mailto:m.malavolta@inrca.it)

**Supplementary figures and tables**


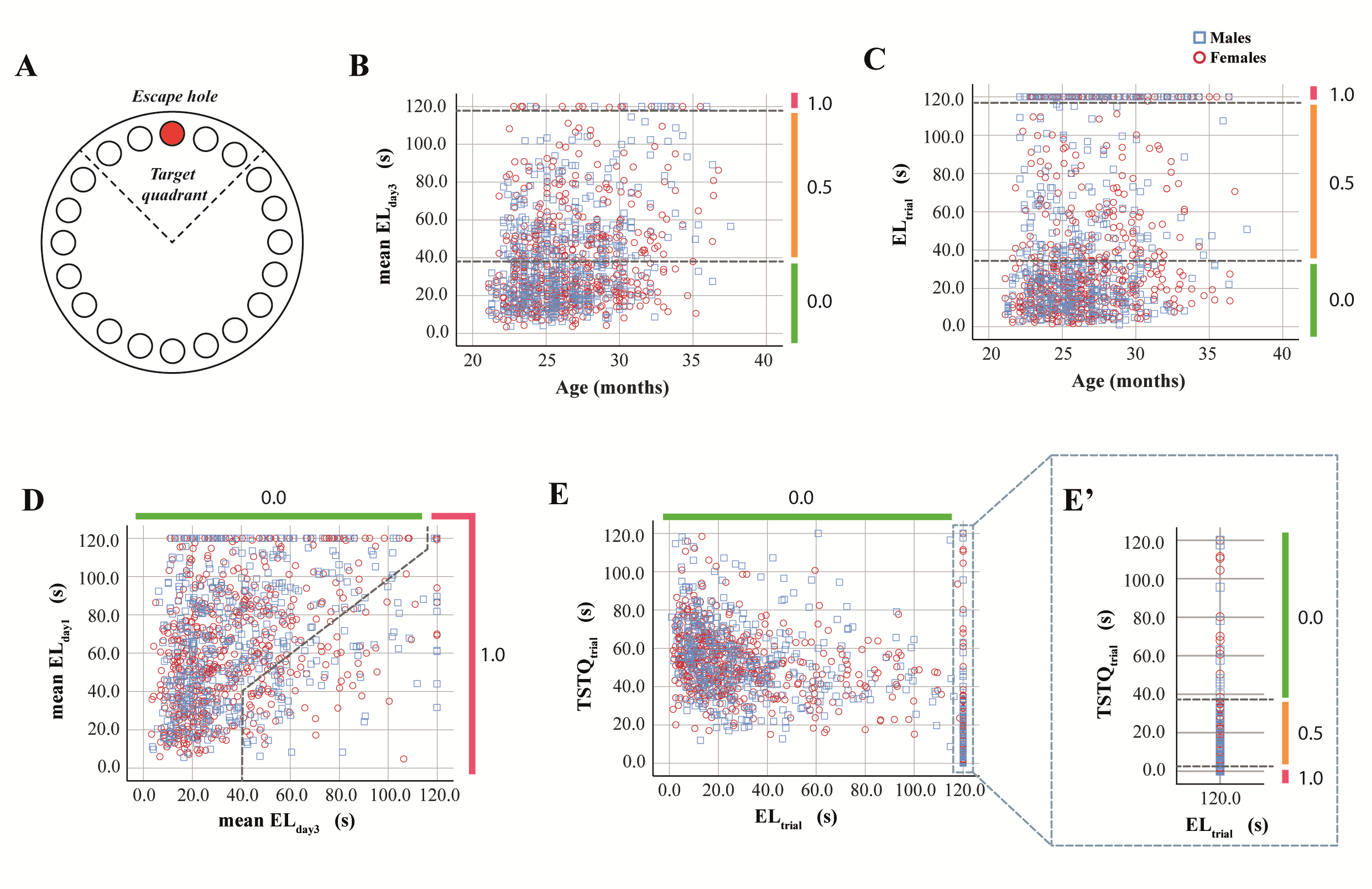


**Fig. S1** The Barnes Maze test.

(A) Schematic illustration of the Barnes maze. The escape hole and the target quadrant are depicted.

(B-E) Identification of the cutoff values for mean EL_d3_ (B), EL_trial_ (C), mean EL_d1-d3_ (D), and TSTQ_trial_ (E) to define a severity rating of cognitive function decline. Dot plots show individual data points for each subject, with blue squares representing male mice and red dots representing female mice. Data are expressed in seconds. Dashed lines indicate defined cutoff points used to assign severity rates, which are shown in the graphs. Severity rates are defined as follows: 1.0 represents the maximum level of severity, indicating a fully altered parameter; 0.5 corresponds to an intermediate level of severity, signifying partial alteration of the parameter; and 0.0 indicates no alteration, representing the minimum severity level. E’ represent a higher magnification of E. Mean EL_d1_: escape latency calculated as the mean of the three repetitions on day 1; mean EL_d3_: escape latency calculated as the mean of the three repetitions on day 3; EL_trial_: escape latency during the acquisition probe trial; TSTQ_trial_: time spent in the target quadrant during the acquisition probe trial.


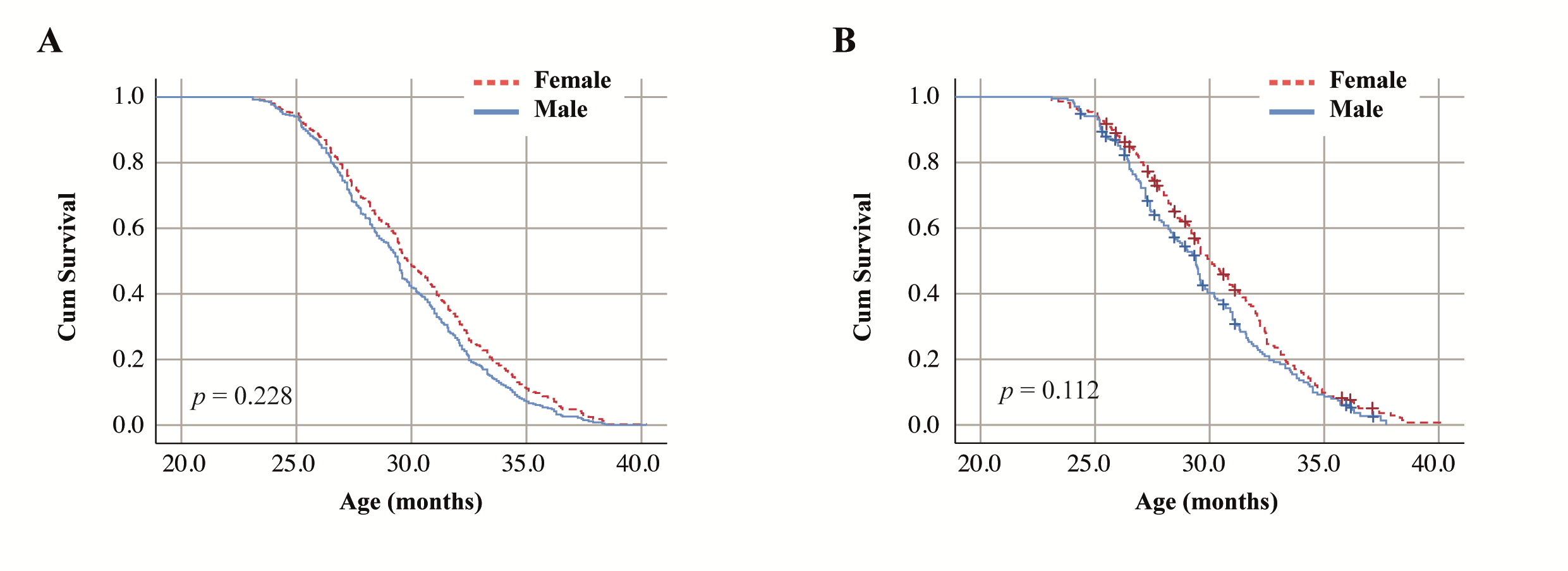


**Fig. S2** Survival curve of the study population.

(A) Cox regression survival curve of male (blue solid line, n = 206) and female (red dashed line, n = 228) C57BL/6J mice, adjusted for age at inclusion and cohort. Mortality occurred when mice died naturally or were euthanized for aging-associated diseases. (B) Kaplan-Meier survival curve (log-rank test) of male (blue line, n = 206) and female (red line, n = 228) C57BL/6J mice. Crosses signify censored events, where the observations ended before natural or humane death (due to organ explantation). Time is expressed in months of age


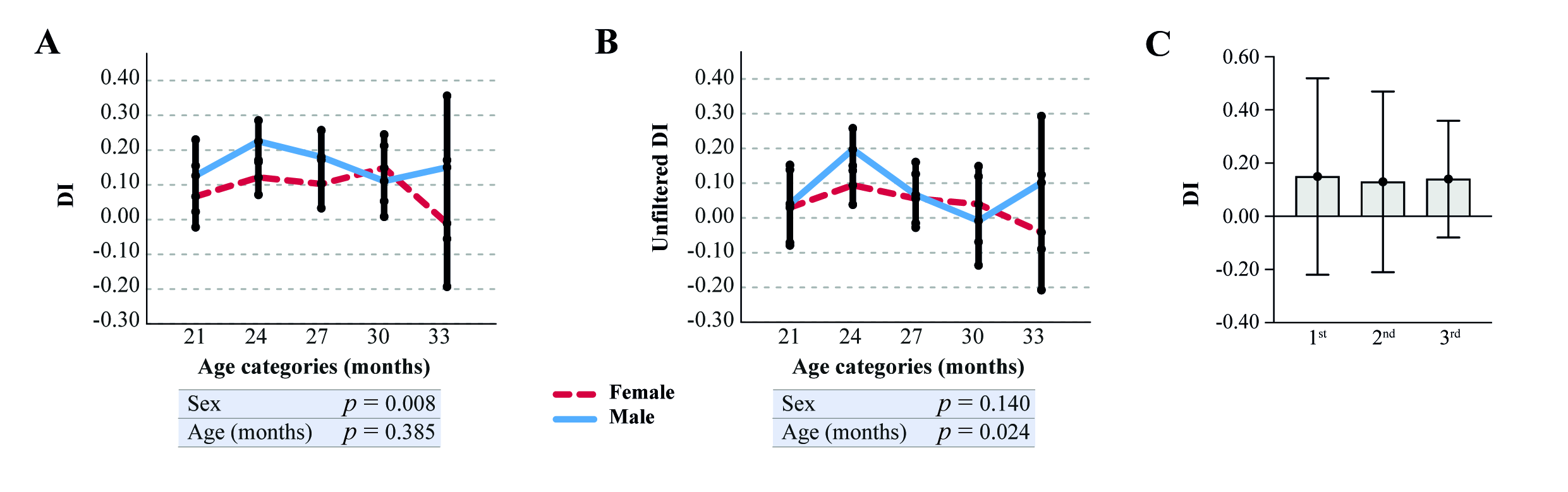


**Fig. S3** Discrimination index (DI) derived from the Novel Object Recognition (NOR) test.

C57BL/6J mice (n = 424) were monitored every three months from the inclusion up to natural death. (A,B) Graphs show the trend with advancing age of the DI (A) and of unfiltered DI (B) both in male (blue solid line) and female (red dashed line) mice. Time is expressed in age categories. The DI was calculated as (TN - TF)/(TN + TF), where TN is the time spent exploring the novel object and TF is the time spent exploring the familiar object. Data were excluded if the total exploration time was less than 20 s (. The unfiltered DI included all data regardless of exploration time. Values are reported as the mean estimates (95% CI) obtained by linear mixed model analysis for longitudinal data using sex, cohort, and age (months) as fixed factors. Test of fixed effects parameters (sex and age) are reported inside the figure. (C) Influence of test repetition on performance parameters derived from the NOR test in a reference population aged 27 months. Data of DI from animals undergoing the test for the first (1^st^) time were compared with those of animals repeating the test for the second (2^nd^) or third (3^rd^) time, with a three-month interval between tests.


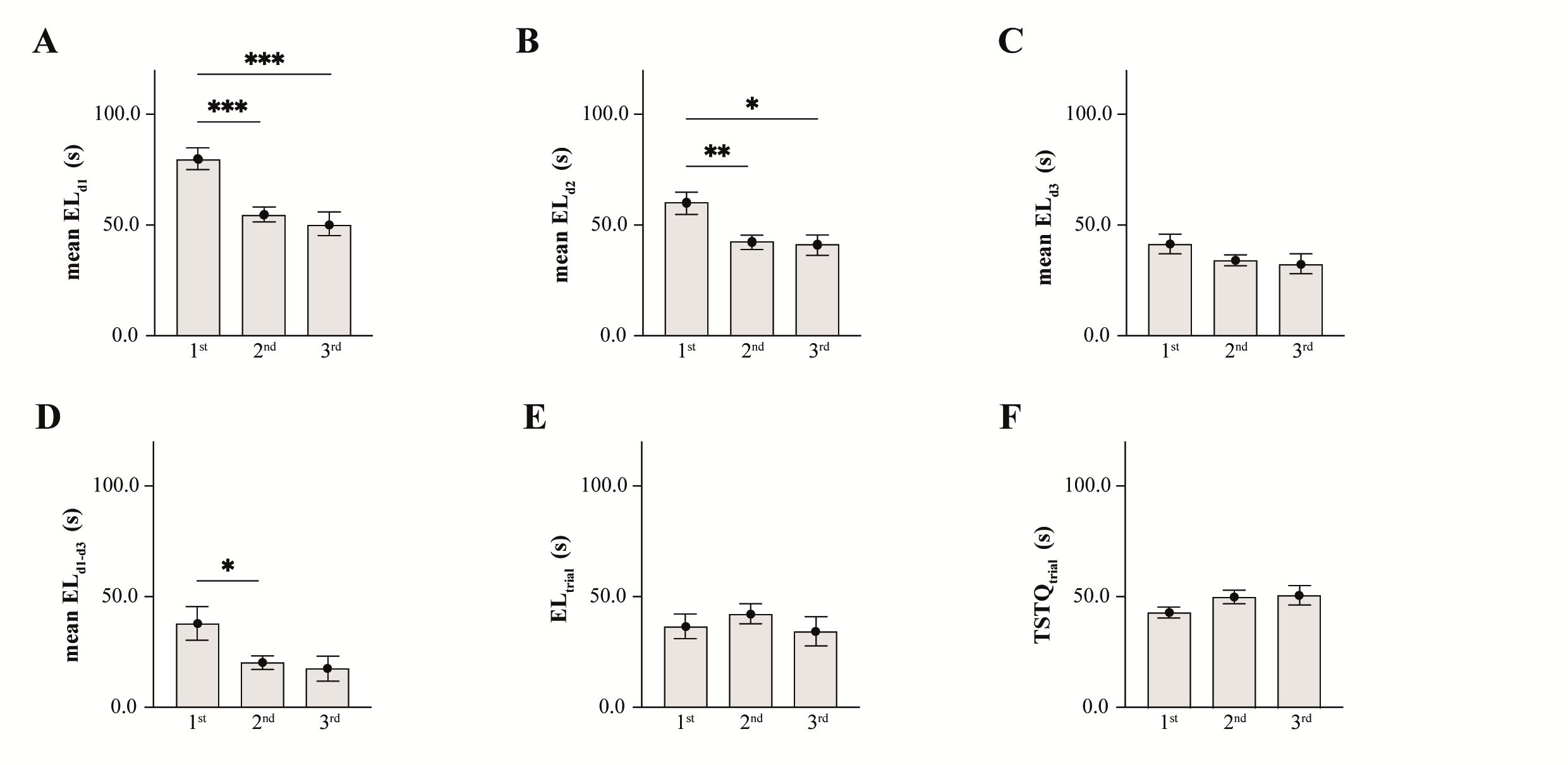


**Fig. S4** Influence of test repetition on performance parameters derived from the Barnes Maze test in a reference population aged 27 months.

Data of mean EL_d1_ (A), mean EL_d2_ (B), mean EL_d3_ (C), mean EL_d1-d3_ (D), EL_trial_ (E), and TSTQ_trial_ (F) from animals undergoing the test for the first (1^st^) time were compared with those of animals repeating the test for the second (2^nd^) or third (3^rd^) time, with a three-month interval between tests. *p < 0.05; **p < 0.01; ***p < 0.001. Mean EL_d1_: escape latency calculated as the mean of the three repetitions on day 1; mean EL_d2_: escape latency calculated as the mean of the three repetitions on day 2; mean EL_d3_: escape latency calculated as the mean of the three repetitions on day 3; mean EL_d1-d3_: mean escape latency on day 1 minus mean escape latency on day 3; EL_trial_: escape latency during the acquisition probe trial; TSTQ_trial_: time spent in the target quadrant during the acquisition probe trial.


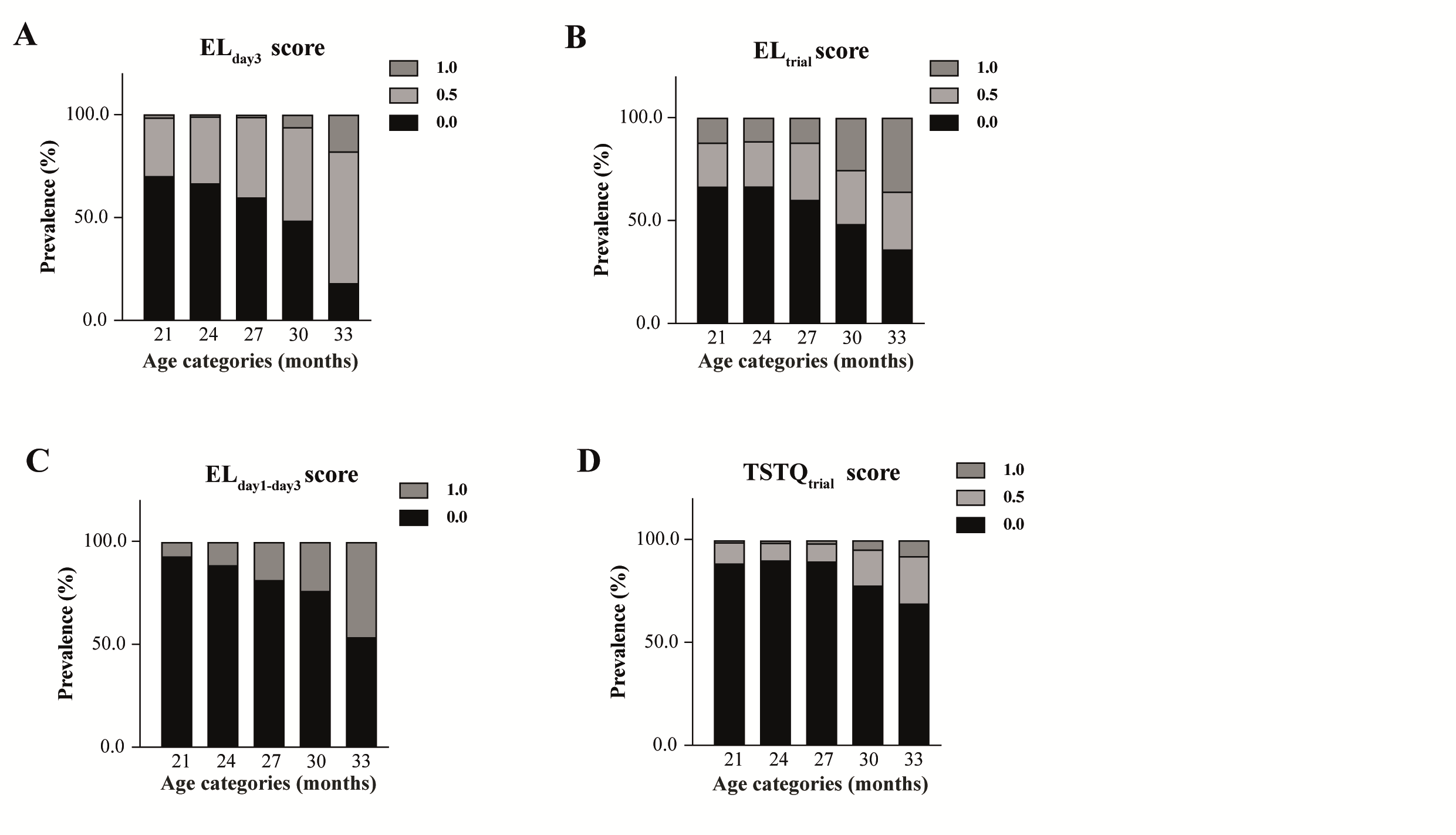


**Fig. S5** Prevalence of severity rates.

The graphs show the prevalence of severity rates at each time-point according to the EL_d3_ score (A), EL_trial_ score (B), EL_d1-d3_ score (C), and TSTQ_trial_ score (D). Severity rates are defined as follows: 1.0 represents the maximum level of severity, indicating a fully altered parameter; 0.5 corresponds to an intermediate level of severity, signifying partial alteration of the parameter; and 0.0 indicates no alteration, representing the minimum severity level. EL_d3_: average of the three escape latency values recorded during the day 3 of the Barnes Maze test; EL_d1-d3_: escape latency on day 1 minus escape latency on day 3; EL_trial_: escape latency during the acquisition probe trial; TSTQ_trial_: time spent in the target quadrant during the acquisition probe trial. Age category 21: 21-23 months of age; age category 24: 24-26 months of age; age category 27: 27-29 months of age; age category 30: 30-32 months of age; age category 33: 33-36 months of age.


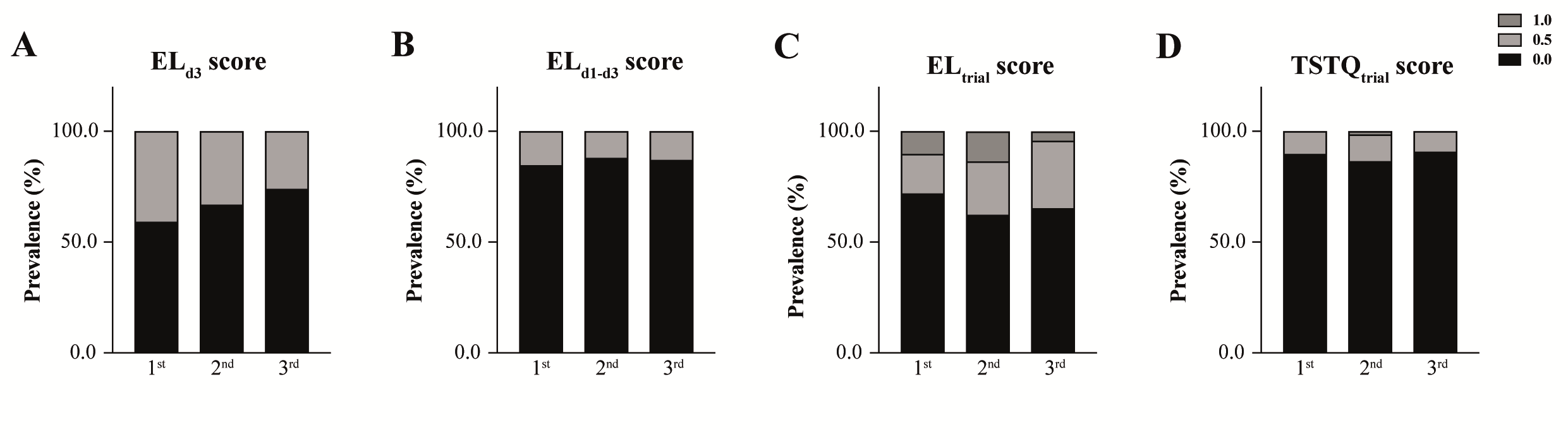


**Fig. S6** Influence of test repetition on the prevalence of severity rates in a reference population aged 27 months.

Data for EL_d3_ score (A), EL_d1-d3_ score (B), EL_trial_ score (C), and TSTQ_trial_ score (D) from animals undergoing the test for the first (1^st^) time were compared with those of animals repeating the test for the second (2^nd^) or third (3^rd^) time, with a three-month interval between tests. Severity rates are defined as follows: 1.0 represents the maximum level of severity, indicating a fully altered parameter; 0.5 corresponds to an intermediate level of severity, signifying partial alteration of the parameter; and 0.0 indicates no alteration, representing the minimum severity level. EL_d3_: escape latency calculated as the mean of the three repetitions on day 3; EL_d1-d3_: escape latency on day 1 minus mean escape latency on day 3; EL_trial_: escape latency during the acquisition probe trial; TSTQ_trial_: time spent in the target quadrant during the acquisition probe trial.


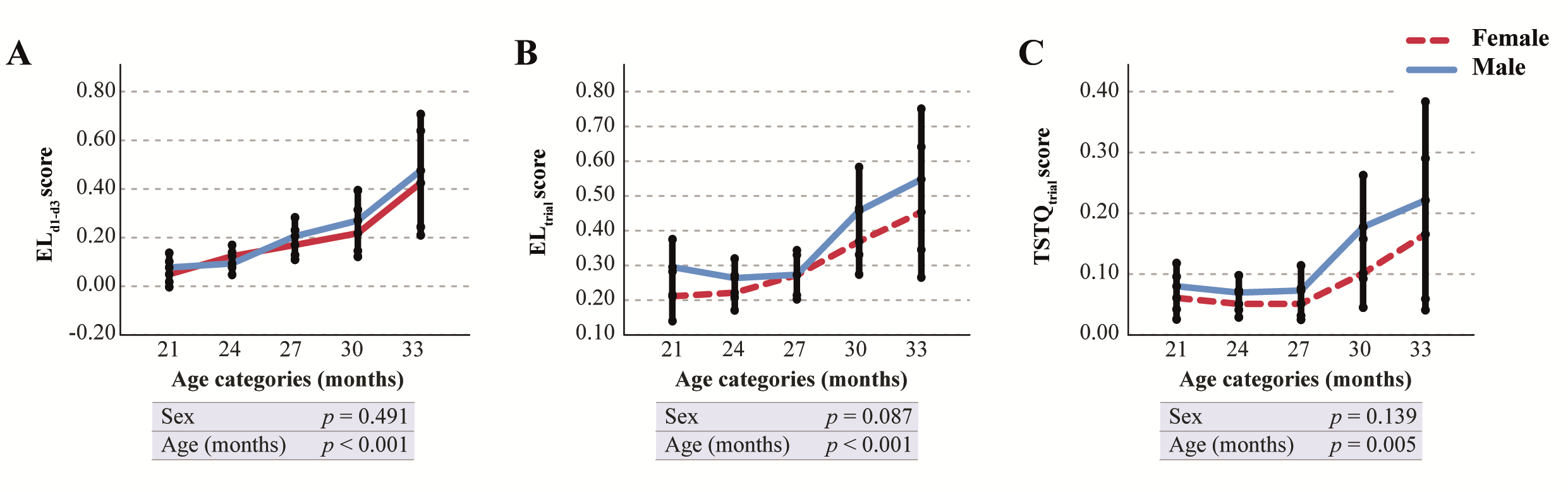


**Fig. S7** Quantitative estimation of the three selected cognitive scores.

C57BL/6J mice (n = 424) were monitored every three months from the inclusion up to natural death. Graphs show the trend with advancing age of the EL_d1-d3_ score (A), EL_trial_ score (B), and TSTQ_trial_ score (C), both in male (blue solid line) and female (red dashed line) mice. Time is expressed in age categories. Values are reported as the mean estimates (95% CI) obtained by generalized linear mixed model analysis for longitudinal data using sex, cohort, and age (months) as fixed factors. Test of fixed effects parameters (sex and age) are reported inside the figure. EL_d1-d3_: escape latency on day 1 minus mean escape latency on day 3; EL_trial_: escape latency during the acquisition probe trial; TSTQ_trial_: time spent in the target quadrant during the acquisition probe trial*.*


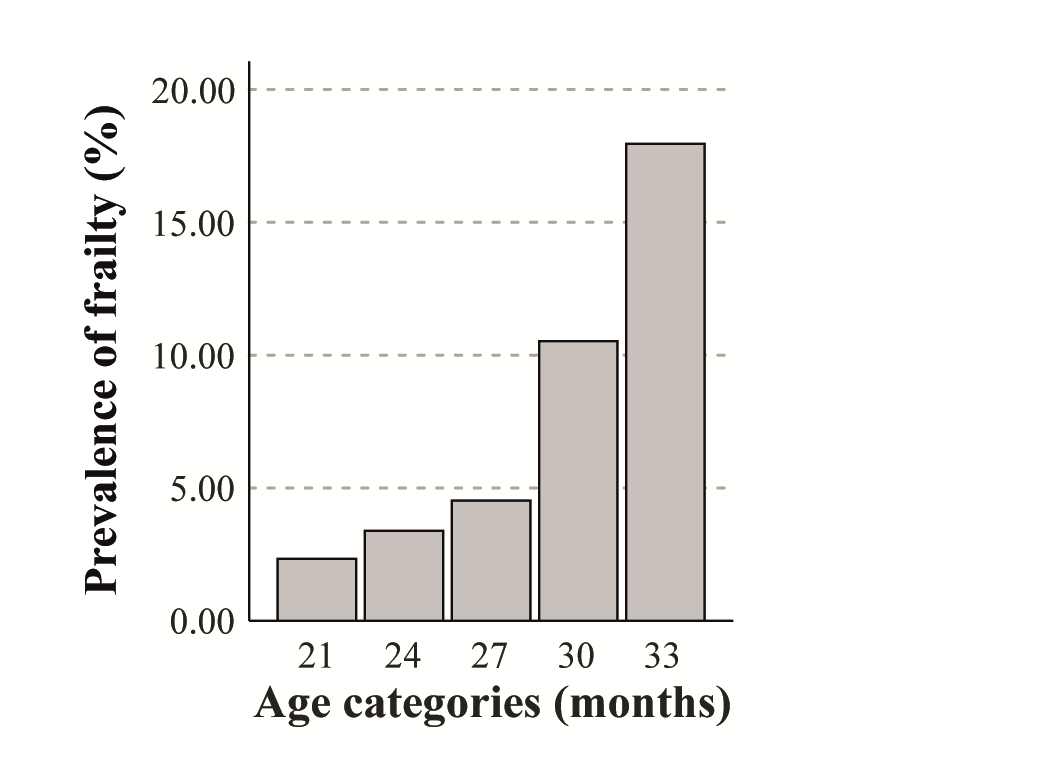


**Fig. S8** Percentage of frailty for the Cognitive Frailty Index (CoFI) in each age category. Cut off for frailty = 0.83.


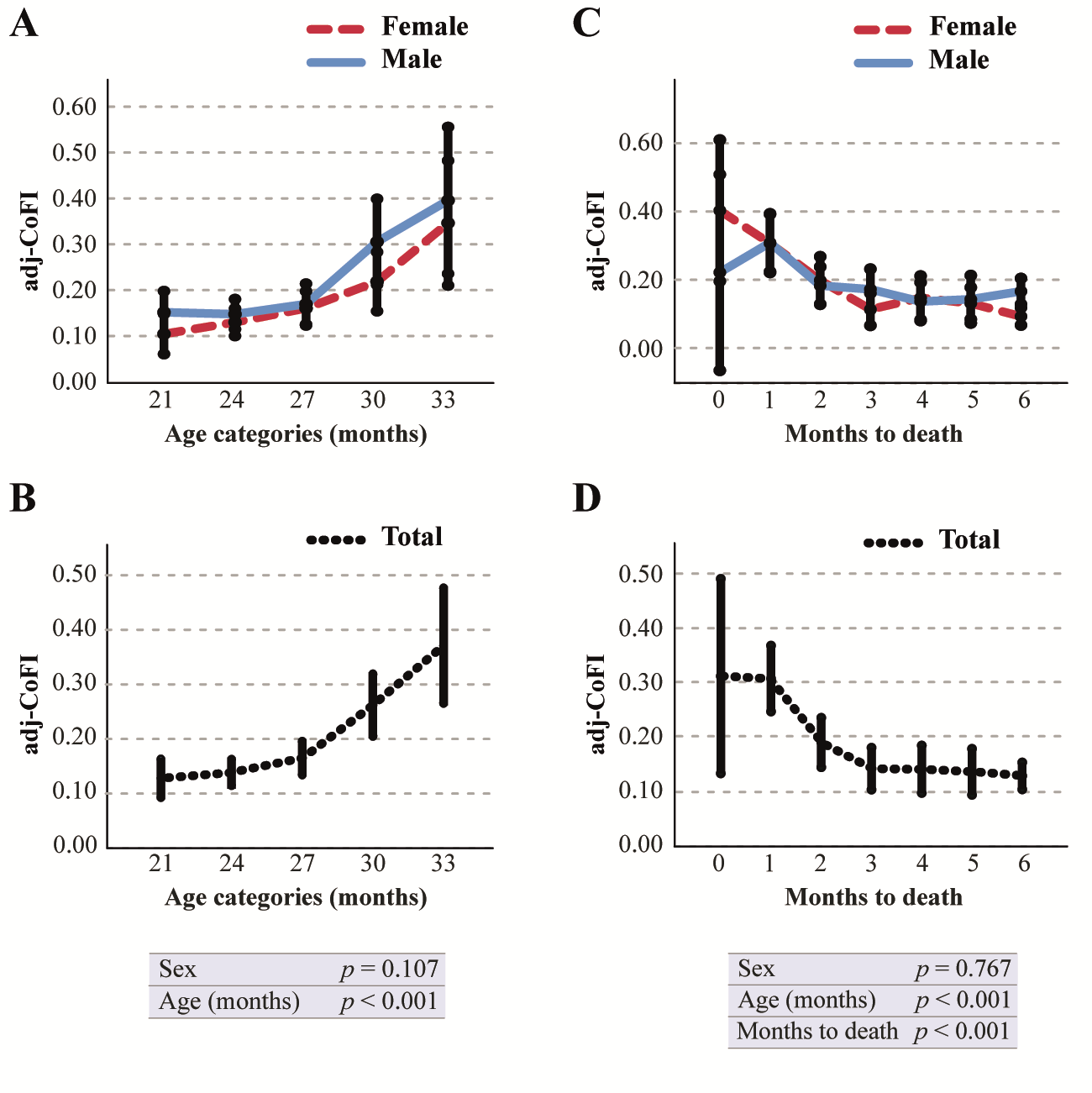


**Fig. S9** Association of the adjusted Cognitive Frailty Index (adj-CoFI) with aging and mortality.

C57BL/6J (n = 424) mice were monitored every three months from the inclusion up to death.

Data of adj-CoFI are reported as a function of age categories (A,B) or months to death (C,D). Time is expressed in age categories (A,C) or as months to death (B,D), respectively. Values are reported as the mean estimates (95% CI) obtained by generalized linear mixed model analysis for longitudinal data using sex, cohort, and age (months) as fixed factors. Tests of fixed effects parameters (sex, age, or months to death) are reported inside the figure. Data from male mice (blue solid line), female mice (red dashed line), or whole population (black dotted line) are presented.

**
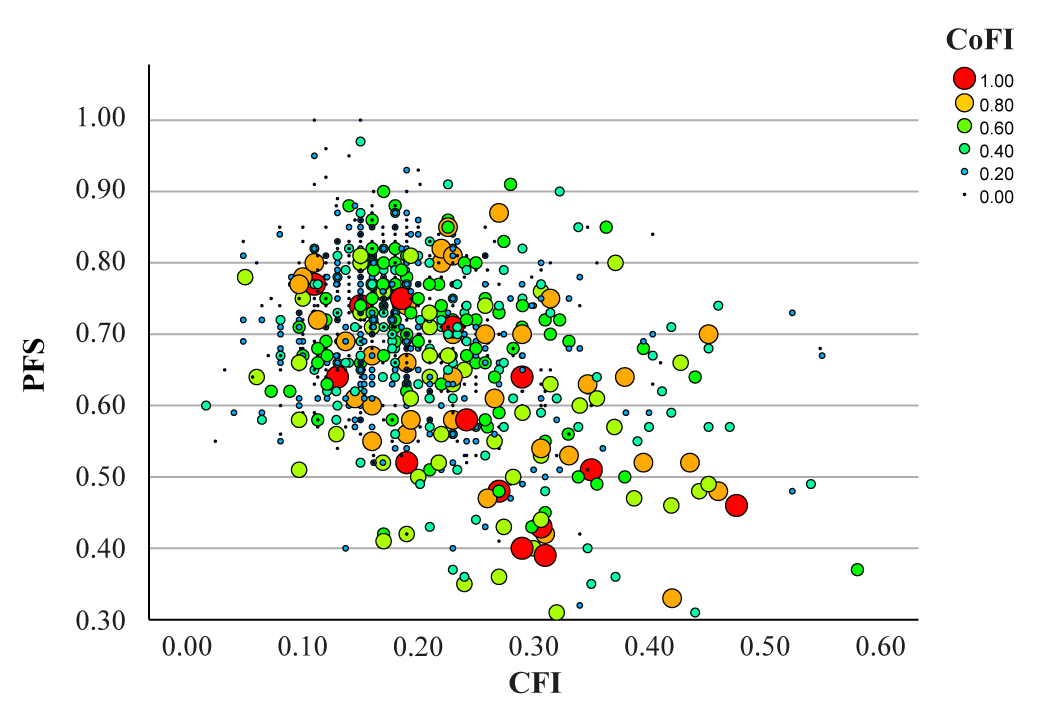
**

**Fig. S10** Bubble plot illustrating the relationships between CFI, PFS, and CoFI.

The plot shows the distribution of PFS as a function of CFI, with the size and color of the dots representing CoFI values. Larger and red dots indicate higher CoFI values, while smaller dots represent lower CoFI values.

Video S1 Example of a mouse with preserved cognitive abilities despite physical impairment

**Table S1** Distribution of mice across cognitive test repetition by age categories.

Each row represents a specific age category (21 months, 24 months…), while the columns indicate the number of mice undergoing the test for the first time (1^st^) and during subsequent repetitions (2^nd^, 3^rd^, etc.). The total at the bottom of each column represents the sum of mice tested at each repetition cycle. The first cognitive measurement for all mice was conducted between 21 and 27 months of age.

|  | **1^st^** | **2^nd^** | **3^rd^** | **4^th^** | **5^th^** | **6^th^** |
| --- | --- | --- | --- | --- | --- | --- |
| ***21 months*** | 158 | 56 | 0 | 0 | 0 | 0 |
| ***24 months*** | 227 | 139 | 67 | 10 | 0 | 0 |
| ***27 months*** | 39 | 94 | 73 | 52 | 7 | 0 |
| ***30 months*** | 0 | 18 | 42 | 22 | 32 | 0 |
| ***33 months*** | 0 | 0 | 6 | 13 | 15 | 5 |
| **TOTAL** | **424** | **307** | **188** | **97** | **54** | **5** |

**Table S2** CoFI computation.

Tertiles identification for EL_d3_ (average of the three escape latency values recorded during the third day of the Barnes Maze test), EL_trial_ (escape latency value recorded during the acquisition probe day), and TSTQ_trial_ (time spent in the target quadrant during the acquisition probe day) in a reference population aged between 21 and 27 months.

|  |  | **EL_d3_** | **EL_trial_** | **TSTQ_trial_** |
| --- | --- | --- | --- | --- |
| ***n*** |  | 799 | 799 | 799 |
| **Mean (s)** |  | 35.5 | 38.8 | 50.1 |
| **Median (s)** |  | 27.8 | 23.6 | 50.6 |
| **Std. Deviation (s)** |  | 24.0 | 37.0 | 22.0 |
| **Minimum (s)** |  | 3.4 | 1.5 | 0.0 |
| **Maximum (s)** |  | 120.0 | 120.0 | 119.9 |
| **Tertiles** | **Second (s)** | 20.6 | 15.4 | 42.2 |
|  | **Third (s)** | 39.4 | 35.6 | 58.2 |

**Table S3** Data of Individual scores comprising the PFS per age category.

The PFS is calculated as the average of five scores: Body Size Score, Strength Score, Endurance Score, Speed Score, and Activity Score. Each score is derived from individual scores, as described in detail in the Materials and Methods of our previously published method (Marcozzi et al. 2023). Data are expressed as Mean ± SD.

|  | **Body Size Score** | **Strength Score** | **Endurance Score** | **Speed Score** | **Activity Score** | **Physical Function Score** |
| --- | --- | --- | --- | --- | --- | --- |
| ***21 months*** | 0.83 ± 0.13 | 0.80 ± 0.10 | 0.70 ± 0.12 | 0.80 ± 0.12 | 0.60 ± 0.23 | 0.75 ± 0.09 |
| ***24 months*** | 0.78 ± 0.15 | 0.77 ± 0.11 | 0.69 ± 0.14 | 0.75 ± 0.15 | 0.56 ± 0.25 | 0.71 ± 0.10 |
| ***27 months*** | 0.70 ± 0.16 | 0.71 ± 0.10 | 0.66 ± 0.14 | 0.74 ± 0.16 | 0.61 ± 0.30 | 0.69 ± 0.11 |
| ***30 months*** | 0.61 ± 0.16 | 0.63 ± 0.13 | 0.57 ± 0.14 | 0.68 ± 0.15 | 0.56 ± 0.25 | 0.63 ± 0.10 |
| ***33 months*** | 0.52 ± 0.17 | 0.56 ± 0.10 | 0.51 ± 0.14 | 0.61 ± 0.15 | 0.54 ± 0.27 | 0.57 ± 0.11 |

.

**Table S4** adj-CoFI computation.

The experimental cohort was divided into three classes (high, medium and low Physical Function Score (PFS)) based on the tertiles of PFS identified in a study population aged 21-27 months. Tertiles identification for EL_d3_ (average of the three escape latency values recorded during the third day of the Barnes Maze test), EL_trial_ (escape latency value recorded during the acquisition probe day), and TSTQ_trial_ (time spent in the target quadrant during the acquisition probe day) in a reference population aged between 21 and 27 months, within the three classes.

|  |  |  | **EL_d3_ (s)** | **EL_trial_ (s)** | **TSTQ_trial_ (s)** |
| --- | --- | --- | --- | --- | --- |
| **High PFS**  **(1.00 – 0.77)** | **Mean** |  | 30.3 | 35.0 | 51.3 |
|  | **Median** |  | 24.4 | 23.0 | 51.3 |
|  | **Tertiles** | **Second** | 18.6 | 14.0 | 42.5 |
|  |  | **Third** | 33.0 | 33.5 | 58.0 |
| **Medium PFS**  **(0.76 – 0.69)** | **Mean** |  | 34.6 | 36.5 | 49.8 |
|  | **Median** |  | 28.1 | 22.0 | 50.7 |
|  | **Tertiles** | **Second** | 20.5 | 14.8 | 41.6 |
|  |  | **Third** | 38.5 | 32.8 | 58.2 |
| **Low PFS**  **(0.68 – 0.00)** | **Mean** |  | 42.1 | 45.1 | 48.9 |
|  | **Median** |  | 32.8 | 26.3 | 49.5 |
|  | **Tertiles** | **Second** | 23.6 | 18.1 | 42.6 |
|  |  | **Third** | 50.9 | 44.8 | 58.5 |

**Table S5** Distribution of the number and percentage of recorded mice per age category.

The frequencies are relative to the Barnes maze data and Novel Object Recognition (NOR) Test. Filtered NOR are the frequency for the NOR after filtering mice for a minimum exploration total time for both objects of 20 s.

|  | **Barnes and Unfiltered NOR** | | **Filtered NOR** | |
| --- | --- | --- | --- | --- |
|  | **Frequency** | **Percentage** | **Frequency** | **Percentage** |
| ***21 months*** | 214 | 19.91 | 98 | 14.69 |
| **Male** | 107 | 21.70 | 50 | 16.20 |
| **Female** | 107 | 18.40 | 48 | 13.40 |
| ***24 months*** | 443 | 41.21 | 278 | 41.68 |
| **Male** | 201 | 40.80 | 129 | 41.70 |
| **Female** | 242 | 41.60 | 149 | 41.60 |
| ***27 months*** | 265 | 24.65 | 181 | 27.14 |
| **Male** | 119 | 24.10 | 81 | 26.20 |
| **Female** | 146 | 25.10 | 100 | 27.90 |
| ***30 months*** | 114 | 10.60 | 81 | 12.14 |
| **Male** | 48 | 9.70 | 37 | 12.00 |
| **Female** | 66 | 11.30 | 44 | 12.30 |
| ***33 months*** | 39 | 3.63 | 29 | 4.35 |
| **Male** | 18 | 3.70 | 12 | 3.90 |
| **Female** | 21 | 3.60 | 17 | 4.70 |

**Table S6** Association between performance parameters derived from the Novel Object Recognition (NOR) and Barnes Maze test and advancing age.

Correlation coefficient and *p*-values analyzed by Spearman correlation are reported. Significant coefficients are highlighted in bold.

|  |  |  | **Age (months)** |
| --- | --- | --- | --- |
| **NOR test** | **DI** | Correlation Coefficient | -0.002 |
|  |  | Sig. (2-tailed) | *p* = 0.955 |
|  | **Unfiltered DI** | Correlation Coefficient | -0.010 |
|  |  | Sig. (2-tailed) | *p* = 0.738 |
| **Barnes test** | **Mean EL_d1_** | Correlation Coefficient | -0.035 |
|  |  | Sig. (2-tailed) | *p* = 0.247 |
|  | **Mean EL_d2_** | Correlation Coefficient | 0.027 |
|  |  | Sig. (2-tailed) | *p* = 0.385 |
|  | **Mean EL_d3_** | Correlation Coefficient | **0.236** |
|  |  | Sig. (2-tailed) | ***p* < 0.001** |
|  | **Mean EL_d1-d2_** | Correlation Coefficient | -0.050 |
|  |  | Sig. (2-tailed) | *p* = 0.099 |
|  | **Mean EL_d1-d3_** | Correlation Coefficient | **-0.217** |
|  |  | Sig. (2-tailed) | ***p* < 0.001** |
|  | **EL_trial_** | Correlation Coefficient | **0.154** |
|  |  | Sig. (2-tailed) | ***p* < 0.001** |
|  | **TSTQ_trial_** | Correlation Coefficient | **-0.081** |
|  |  | Sig. (2-tailed) | ***p* = 0.008** |

**Table S7** Description of the study population.

Short-lived mice (n = 98) died within 27 months; long-lived mice (n = 65) died after 33 months. The age at death was estimated with Kaplan-Meier.

|  | **SHORT-LIVED** | **LONG-LIVED** |
| --- | --- | --- |
| ***n*** | 98 | 65 |
| **Male** | 55 | 27 |
| **Female** | 43 | 38 |
| **Age at enrollment (months)** | 23.44 ± 1.35 | 23.69 ± 1.30 |
| **Male** | 23.53 ± 1.35 | 23.56 ± 1.30 |
| **Female** | 23.32 ± 1.37 | 23.78 ± 1.31 |
| **Age at death**  **(months)** | 26.09 ± 0.07 | 36.65 ± 0.25 |
| **Male** | 26.11 ± 0.09 | 37.03 ± 0.33 |
| **Female** | 26.06 ± 0.11 | 35.78 ± 0.24 |

**Table S8** Crosstabulation between frail subject for Cognitive Frailty (CoFI) and frail subject for PFS, showing the distribution of cases across categories. Fisher’s exact test was used to assess the statistical significance of the association between the two variables. Values in each cell represent the count. p-values indicate the significance level of the observed associations. Significant coefficients are highlighted in bold.

|  |  |  | **PFS** | | **Total** | **Fisher’s Exact *p*** |
| --- | --- | --- | --- | --- | --- | --- |
|  |  |  | **Healthy** | **Frail** |  |  |
| ***21 months*** | **CoFI** | **Healthy** | 202 | 7 | 209 | 1.000 |
|  |  | **Frail** | 5 | 0 | 5 |  |
|  | **Total** | | 207 | 7 | 214 |  |
| ***24 months*** | **CoFI** | **Healthy** | 405 | 23 | 428 | **0.009** |
|  |  | **Frail** | 11 | 4 | 15 |  |
|  | **Total** | | 416 | 27 | 443 |  |
| ***27 months*** | **CoFI** | **Healthy** | 225 | 28 | 253 | **0.043** |
|  |  | **Frail** | 8 | 4 | 12 |  |
|  | **Total** | | 233 | 32 | 265 |  |
| ***30 months*** | **CoFI** | **Healthy** | 82 | 20 | 102 | 0.275 |
|  |  | **Frail** | 8 | 4 | 12 |  |
|  | **Total** | | 90 | 24 | 114 |  |
| ***33 months*** | **CoFI** | **Healthy** | 21 | 11 | 32 | 0.396 |
|  |  | **Frail** | 3 | 4 | 7 |  |
|  | **Total** | | 24 | 15 | 39 |  |

**Table S9** Association of cognitive frailty, physical frailty, and their combination, defined by CoFI and PFS thresholds, with one-month mortality. Animals were classified into: cognitively frail only (frail according to CoFI but not PFS), physically frail only (frail according to PFS but not CoFI), and frail in both domains (frail according to both CoFI and PFS). The table shows that frailty in both domains combined is a stronger predictor of mortality than either domain alone.

| **Frail status** | **Coefficient*** | ***p*** **value** | **95% Confidence Interval** | | **Exp(coefficient)** | **95% Confidence Interval for exp(coefficient)** | |
| --- | --- | --- | --- | --- | --- | --- | --- |
|  |  |  | **Lower** | **Upper** |  | **Lower** | **Upper** |
| **Cognitive Frailty**  **(CoFI > 0.83)** | 1.49 | **0.002** | 0.54 | 2.44 | **4.44** | 1.72 | 11.47 |
| **Physical Frailty**  **(PFS < 0.55)** | 2.16 | **< 0.001** | 1.61 | 2.72 | **8.70** | 4.98 | 15.18 |
| **Cognitive and Physical Frailty**  **(CoFI > 0.83 and PFS < 0.55)** | 2.74 | **< 0.001** | 1.65 | 3.84 | **15.54** | 5.21 | 46.31 |

* Coefficient table of fixed effects parameters obtained by fitting the generalized linear mixed model with a binomial distribution and logit link function. Significant coefficients are highlighted in bold.

**Table S10** Crosstabulation between frail subject for adj-CoFI and frail subject for PFS, showing the distribution of cases across categories. Fisher’s exact test was used to assess the statistical significance of the association between the two variables. Values in each cell represent the count. p-values indicate the significance level of the observed associations.

|  |  |  | **PFS** | | **Total** | **Fisher’s Exact *p*** |
| --- | --- | --- | --- | --- | --- | --- |
|  |  |  | **Healthy** | **Frail** |  |  |
| ***21 months*** | **adj-CoFI** | **Healthy** | 203 | 7 | 210 | 1.000 |
|  |  | **Frail** | 4 | 0 | 4 |  |
|  | **Total** | | 207 | 7 | 214 |  |
| ***24 months*** | **adj-CoFI** | **Healthy** | 406 | 24 | 430 | **0.038** |
|  |  | **Frail** | 10 | 3 | 13 |  |
|  | **Total** | | 416 | 27 | 443 |  |
| ***27 months*** | **adj-CoFI** | **Healthy** | 226 | 29 | 255 | 0.106 |
|  |  | **Frail** | 7 | 3 | 10 |  |
|  | **Total** | | 233 | 32 | 265 |  |
| ***30 months*** | **adj-CoFI** | **Healthy** | 82 | 20 | 102 | 0.275 |
|  |  | **Frail** | 8 | 4 | 12 |  |
|  | **Total** | | 90 | 24 | 114 |  |
| ***33 months*** | **adj-CoFI** | **Healthy** | 21 | 11 | 32 | 0.396 |
|  |  | **Frail** | 3 | 4 | 7 |  |
|  | **Total** | | 24 | 15 | 39 |  |

**Table S11** Association of cognitive frailty, physical frailty, and their combination, defined by adj-CoFI and PFS thresholds, with one-month mortality. Animals were classified into: cognitively frail only (frail according to adj-CoFI but not PFS), physically frail only (frail according to PFS but not adj-CoFI), and frail in both domains (frail according to both adj-CoFI and PFS).The table shows that frailty in both domains combined is a stronger predictor of mortality than either domain alone.

| **Frail status** | **Coefficient*** | ***p* value** | **95% Confidence Interval** | | **Exp(coefficient)** | **95% Confidence Interval for exp(coefficient)** | |
| --- | --- | --- | --- | --- | --- | --- | --- |
|  |  |  | **Lower** | **Upper** |  | **Lower** | **Upper** |
| **Cognitive Frailty (adj-CoFI > 0.83)** | 1.62 | **< 0.001** | 0.67 | 2.57 | **5.05** | 1.95 | 13.03 |
| **Physical Frailty (PFS < 0.55)** | 2.17 | **< 0.001** | 1.62 | 2.73 | **8.79** | 5.07 | 15.25 |
| **Cognitive and Physical Frailty (adj-CoFI > 0.83 and PFS < 0.55)** | 2.83 | **< 0.001** | 1.65 | 4.01 | **16.89** | 5.19 | 54.98 |

* Coefficient table of fixed effects parameters obtained by fitting the generalized linear mixed model with a binomial distribution and logit link function. Significant coefficients are highlighted in bold.
